# Influence of Storage Conditions on Stability of Phenolic Compounds and Antioxidant Activity Values in Nutraceutical Mixtures with Edible Flowers as New Dietary Supplements

**DOI:** 10.3390/antiox12040962

**Published:** 2023-04-19

**Authors:** Martina Mrázková, Daniela Sumczynski, Jana Orsavová

**Affiliations:** 1 Department of Food Analysis and Chemistry, Tomas Bata University in Zlín, Vavrečkova 5669, 760 01 Zlín, Czech Republic; 2 Language Centre, Tomas Bata University in Zlín, Štefánikova 5670, 760 01 Zlín, Czech Republic

**Keywords:** phenolic compound, flavonoid, antioxidant activity, nutraceutical mixture, edible flower, non-traditional flake, dietary supplement, cereal

## Abstract

This paper investigates the effects of storage conditions on the stability of phenolics and their antioxidant activities in unique nutraceutical supplements containing non-traditional cereal flakes, edible flowers, fruits, nuts, and seeds. Significant total phenolic content (TPC) of 1170–2430 mg GAE/kg and total anthocyanin content (TAC) with the values of 322–663 mg C3G/kg were determined with the highest TPC content established in free phenolic fractions. The most notable declines in TPC (by 53%), TAC (by 62%), phenolics (e.g., glycosylated anthocyanins by 35–67%), and antioxidant activity (by 25% using DPPH) were established in the presence of sunlight at 23 °C followed by the storage at 40 °C. Quercetin, rutin, peonidin, pelargonidin, *p-*coumaric, ellagic, and *p-*hydroxybenzoic acids were identified as the least stable phenolics when exposed to sunlight. Furthermore, glycosylated forms of anthocyanins demonstrated a greater stability when compared with anthocyanidins. The mixtures considerably eliminated ABTS and DPPH radicals. In all samples, water-soluble substances showed a higher antioxidant activity than lipid-soluble substances with the main contributors in the following order: delphinidin-3-glucoside (*r* = +0.9839) > *p-*coumaric > gallic > sinapic > *p-*hydroxybenzoic acids > delphinidin > peonidin and malvidin (*r* = +0.6538). Gluten-free nutraceutical mixtures M3 (containing red rice and black quinoa flakes, red and blue cornflowers, blueberries, and barberries) and M4 (containing red and black rice flakes, rose, blue cornflower, blueberries, raspberries, and barberries) were evaluated as the least stable under all storage conditions although they showed considerable phenolic concentrations. Phenolic contents and antioxidant activity of the nutraceutical mixtures were the highest at 23 °C without the presence of sunlight with the most stable M1 nutraceutical mixture (containing oat and red wheat flakes, hibiscus, lavender, blueberries, raspberries, and barberries).

## 1. Introduction

Breakfast cereals should provide principal nutrients required for human health. They supply energy, carbohydrates, protein, dietary fiber, vitamins, minerals, and antioxidants [1]. Different valuable biological properties of these compounds have been already documented since the diet incorporates whole grains rich in phenolic compounds which exhibit antioxidant activity which reduces the risk of various chronic diseases. Additionally, a regular intake of cereal grains with colored coating layers accompanied by fruits can help prevent headache, reduce the risk of colon cancer, obesity, heart diseases and the onset of Alzheimer’s disease, as well as regulate blood sugar level [2,3,4,5,6,7,8]. Development of several common chronic degenerative diseases is considered to be initiated by reactive oxygen and nitrogen species responsible for the oxidative stress in cells [6,9]. Generally, oxidative stress occurs when free radicals are present at higher amounts and thus remain unpaired as the antioxidant defense cannot deal with their imbalance. Antioxidant compounds decrease such oxidative stress by pairing reactive oxygen free electrons and include phenolic compounds; their beneficial antioxidant properties allowed them to be considered as nutraceuticals. A great focus has been dedicated to developing protocols of the extractions of polyphenols from multiple herb species to produce supplements. However, side effects of regular intake of high doses of these phenolic supplements, such as liver disorder or stroke, have been reported as well [6,10]. What is more, consumption of isolated individual polyphenolic compounds may not provide the same benefits as those observed in epidemiological studies [10]. Therefore, innovative nutraceutical mixtures containing food ingredients rich in antioxidants, mainly polyphenolic substances, seem to be a novel solution to this issue and breakfast cereals may be such a promising product. Breakfast cereals are consumed the most in the UK (approximately 8 kg per year and person), followed by the USA, Australia, and Canada. Their consumption in Europe is only about 1.5 kg per year per person [11]. One type of RTE cereal mixture is muesli, where grains are modified by flaking or extrusion and other components are added, particularly dried fruits, nuts, and substances modifying taste, smell, and consistency [12].

A healthy lifestyle has been increasingly promoted; therefore, new foods are being developed. From a nutritional point of view, pigmented grains can be considered as healthy foods containing fiber and phenolic antioxidants. Gluten-free grains prevail in the celiac patients’ diet [13]. Celiac disease is an immune-mediated disease characterized by malabsorption of nutrients in the gastrointestinal tract. Commonly consumed cereals and pseudocereals may meet the requirements of a gluten-free diet which is otherwise generally poor in nutrients such as polyphenol antioxidants [14]. Therefore, the production of value-added pigmented gluten-free flakes with high antioxidant activity values may enhance the quality of celiac patients’ diet. Non-traditional pigmented flakes contain high amounts of phenolic acids and flavonoids, including gallic, vanillic, sinapic, *p-*coumaric, cinnamic and ellagic acids, rutin, epigallocatechin, and catechin. Most of them have been evaluated as the main contributors to the antioxidant activity in free, soluble bound, or insoluble bound phenolic fractions [15]. In addition, there are naturally occurring color substances in pigmented grains belonging to the anthocyanins, such as cyanidin-3-glucoside, delphinidin-3-glucoside, cyanidin-3-rutinoside, and their anthocyanidins. What is more, these have been reported to possess free radical scavenging activity [2]. Apart from pigmented flakes, forest and berry fruits are a valuable source of antioxidants as well. Hence berries have been the subject of a number of health-related studies revealing the fact they excel with their levels of carotenoids and vitamin C and they contain a wide profile of polyphenolic compounds, especially anthocyanins, flavones, and flavonols [6,7,16,17].

The visual food appeal has become another required food attribute and may be enhanced by edible flowers even though they are primarily purchased as a garnishing ingredient [18]. Recently, edible flowers may become a suitable component of breakfast cereals as a promising source of antioxidants and nutrients with high antioxidant activity features [19,20]. They contain a diverse range of bioactive compounds, including phenolic substances, vitamin C, carotenes, and xanthophylls which can control oxidative stress [18,20,21,22]. When compared with fruits, they may contain higher concentrations of polyphenolics. Providing edible flowers and herbs are carefully processed, for example by drying, polyphenolic substances are incorporated in up to two orders of magnitude higher concentrations (referred to as dry weight) [23]. Therefore, they could positively affect total phenolic content and antioxidant activity values of food even in smaller proportions. So far, many industrial applications of edible flowers have focused on salads, herbal infused drinks, candied flowers, and gastronomic meals [24]. However, the promising hypothesis that the addition of edible flowers to cereal mixtures will enhance their antioxidant values has been disregarded as of yet. Considering these potential nutritional benefits, Tomas Bata University in Zlín (Czech Republic) has subjected the cereal nutraceutical food mixture employing edible flowers to a patent license [25].

What certainly deserves the attention is their stability and suitable storage conditions [26]. The product must preserve its chemical, physical, microbiological, and sensorial characteristics and, where appropriate, comply with any label declaration of nutritional information when stored according to the recommended protocols [27,28]. Considering the development of new nutraceutical mixtures, it is necessary to know not only their shelf life from the point of view of basic nutritional values, such as the content of lipids, proteins, and carbohydrates, but also the changes in the content of biologically active substances [27]. Many experiments have been devoted to the stability of polyphenolic substances in the presence or absence of sunlight, oxygen, and under the influence of different temperatures during both the storage and various thermal treatments, such as blanching, cooking, roasting, and freezing. Polyphenolic substances have been shown to degrade more when exposed to sunlight, temperatures higher than 40 °C, oxygen, and high humidity [29,30,31]. For these reasons, nutraceutical mixtures were packed using vacuum technology in this experiment. Vacuum technology is commonly used in food packaging as its application in food systems can offer many advantages in various areas [28,32]. Taking these facts into consideration, this study aims to prepare nutraceutical mixtures with edible flowers and non-traditional pigmented flakes, which were prepared by using a hydrothermal treatment of grains with a subsequent flaking.

The objective of this study was to determine the concentrations of individual phenolics and to test the stability of newly developed patented nutraceutical mixtures with edible flowers, non-traditional pigmented flakes, fruits, nuts, and seeds. What is more, this study also evaluates the stability of phenolics under the appropriate storage conditions for a 1-year shelf life as follows: storage without the presence of sunlight at 23 °C, storage in the presence of sunlight at 23 °C and finally, storage in a thermostatic device at 40 °C. To evaluate this stability, free, soluble bound, and insoluble bound phenolics were monitored to observe possible changes in their concentrations and antioxidant activity values. Finally, appropriate correlations between phenolic compounds and antioxidant activity values were calculated.

## 2. Materials and Methods

### 2.1. Ingredient Composition of Nutraceutical Mixtures

Non-traditional flakes of red and black rice (*Oryza sativa* L.) and quinoa (*Chenopodium quinoa* Willd.) seeds were prepared using a Combi-Star mill grinder equipped with a flake roller (Waldner Biotech, Lienz, Austria). The appropriate colored grains were purchased in the local shop in Zlín (Czech Republic). Whole grains were cooked in water (80 °C) under thermometer control for 25–30 min. After being allowed to be conditioned at room temperature for 30 min, they were finalized using a Combi-Star flake roller at a temperature of 23 °C. Finalized flakes were dried at 30 °C and their moisture content decreased by less than 14% [12]. The thickness of colored rice and quinoa flakes was 0.30 and 0.10 mm, respectively. Further types of flakes, such as oat (*Avena sativa* L.), rye (*Secale cereale* L.), kamut (*Triticum turgidum* subsp. *turanicum*), red wheat (*Triticum aestivum* var. *milturum*), white teff (*Eragrostis tef* L.), and white quinoa flakes (*Chenopodium quinoa* Willd.), were purchased in food shops in Zlín (Czech Republic) in the amount of seven packages of 400–500 g. Dried edible flowers of hibiscus *(Hibiscus sabdariffa)*, lavender (*Lavandula angustifolia)*, rose *(Rosa centifolia),* mallow (*Malva sylvestris* var. *mauritiana)* and red and blue cornflower *(Centaurea cyanus*) were supported by Sonnentor and Oxalis companies (Čejkovice, Slušovice, Czech Republic) in the amount of three packages of 200–300 g. Additional ingredients, including hemp seeds, almonds, dried or lyophilized raspberry, barberry, apple, blueberry, strawberry, cherry, and goldenberry, were purchased in local shops (Zlín, Czech Republic) in the amount of five packages of 50–300 g or supported by Vita Cup (K-Servis, Prague, Czech Republic).

### 2.2. Preparation of Nutraceutical Mixtures with Edible Flower

**Figure 1:**
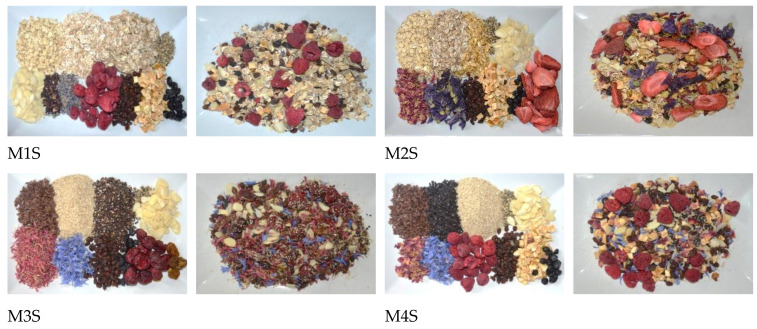
Non-traditional nutraceutical mixtures with edible flowers according to recipe presented in Table 1.

Four nutraceutical mixtures containing edible flowers (samples M1S–M4S) were formulated for the purposes of this study and subjected to a patent license [25]. They were prepared by applying the mixing ratio of 60% of flakes to 40% of lyophilized fruits, nuts, and dried edible flowers in the amount of 5 kg for each sample. Two out of four mixtures were designed gluten-free (M3S and M4S samples) [33]. Since oat flakes were not declared as gluten-free food, they were included in the first two mixtures (M1S and M2S samples) containing gluten. Detailed specific composition of these proposed nutraceutical mixtures protected by a patent license is provided in Table 1 and Figure 1. Each nutraceutical mixture (5 kg) was immediately divided into 48 equal medium-vacuum sealable bags of 100 g each. The bag was created from two bonded layers with an outer polyamide shell and inner polyethylene shell of a size of 20 cm × 30 cm (PolyScience, Frýdlant, Czech Republic). Afterwards, it was hermetically sealed with a vacuum packaging machine (Turbovac, London, UK).

### 2.3. Storage Conditions for Nutraceutical Mixtures with Edible Flowers

According to the current EU legislation, the shelf life, known as a “date of minimum durability”, is applicable to products that are not perishable and do not constitute an immediate danger to human health [34]. Taking this into account, appropriate storage conditions were applied to monitor the stability of phenolic compounds and changes in antioxidant activity values under various conditions [33]:

(a) One part of nutraceutical cereal mixtures (8 × 100 g of each cereal mixture) was stored in an air-conditioned room in the absence of sunlight at a temperature of 23 ± 2 °C. The samples were denoted as M1D to M4D.
(b) Another part of the nutraceutical cereal samples (8 × 100 g of each cereal mixture) was stored in an air-conditioned laboratory under sunlight at a temperature of 23 ± 2 °C. These samples were labeled as M1L to M4L.
(c) The last set of nutraceutical cereal samples (8 × 100 g of each cereal mixture) was placed into a BT120 type incubator (BMT Medical Technology, Brno, Czech Republic) without the presence of sunlight. To test possible variations in the climatic conditions different from those in Central Europe was the reason to set the temperature to 40 °C. The samples were denoted as M1T to M4T.

Moreover, the content of biologically active substances and their antioxidant activity values were monitored at the beginning of the storage trial and after a one-year storage period to evaluate their stability. The samples at the start of the storage experiment were labeled as M1S to M4S. Representative ground samples (100 g) were not stored for more than a week prior to the analysis and each sample was analyzed three or eight times. Dry matter and ash content of the individual sample were assessed to express the results in dry weight values [35,36].

### 2.4. Extraction of Free, Soluble Bound, and Insoluble Bound Phenolics

Individual fractions of free, soluble, and insoluble bound phenolics were extracted using the method reported by [37] with a few minor modifications. The sample of ground nutraceutical mixtures weighing 1.5 g was treated twice with 15 mL of 80% aqueous methanol in a sonic bath at 30 °C for 1 h. Obtained supernatants were mixed and left to evaporate carefully using the water bath. The evaporated part of the sample was then dissolved in 9 mL of 80% methanol with 1 mL of ethyl acetate to gain the extract of free phenolics. To obtain soluble bound phenolic fractions, the 1.5 g sample of ground nutraceutical mixtures was treated twice with 15 mL of 80% aqueous methanol in a sonic bath at 30 °C for 1 h. As in the case of free phenolics, the supernatants obtained were mixed and left to evaporate carefully using the water bath. Consequently, dried extracts were dissolved in 20 mL of 0.1 M NaOH in a nitrogen atmosphere and extracted for 4 h using the magnetic stirrer. Afterwards, the samples were acidified with 6 M HCl until the pH of the sample reached the range of 3 to 4 and then were centrifuged at 1300× *g* for 15 min (Velocity 13μ, Dynamica Scientific Ltd., Livingston, UK). Considering insoluble bound phenolics, the solid residues obtained after the extraction of free fractions were rinsed with distilled water and mixed with 20 mL of 0.1 M NaOH. The extraction was performed in the nitrogen atmosphere for 4 h with the sample being mixed using a magnetic stirrer. Finally, the extracts were acidified with 6 M HCl until the pH of the mixture reached the values between 3–4 and then were centrifuged at 12,300× *g* for 15 min.

### 2.5. Determination of Total Phenolic Content

Total phenolic contents (TPC) were measured colorimetrically using Folin-Ciocalteu reagent [38]. Briefly, 100 µL of the phenolic extract were added to the mixture of 5 mL of redistilled water and 0.5 mL of Folin-Ciocalteu reagent. After a 5-min equilibration, the solution was neutralized with 1.5 mL of 20% NaCO_3_ and the sample was mixed thoroughly. After being allowed to rest for 30 min, the absorbance of the mixture was recorded at 765 nm with a Lambda 25 spectrophotometer (Perkin Elmer, Waltham, MA, USA). TPC values were calculated using the standard gallic acid curve and expressed as mg of gallic acid equivalent per kg of the sample in dry weight (mg GAE/kg).

### 2.6. Determination of Individual Phenolics Using HPLC

The profile of individual flavonoids and phenolic acids in appropriate extracted fractions was measured using the HPLC system consisting of Thermo Scientific DionexUltiMate 3000 Diode Array Detector type DAD-3000RS, Ultimate 3000 rapid separation autosampler, binary pump HPG-3xRS, and solve selector valve HPG-3400RS (Waltham, MA, USA). Data signals were processed on PC running LC Chromeleon^TM^ 7.2 chromatography data system (Thermo Scientific, Waltham, MA, USA). The phenolic profile was determined according to [39] with the following chromatographic conditions: analytical separation was performed using a Kinetex C18 column (150 × 4.6 mm; 5 μm) (Phenomenex, Torrance, CA, USA). During the analysis, 10 µL of the extract was loaded and injected by an autosampler and eluted through the column with a mobile gradient phase consisting of A (redistilled water/acetic acid, 99:1) and B (redistilled water/acetonitrile/acetic acid, 67:32:1) with a flow rate of 1 mL/min. The solvent flow rate was maintained for the total running time of 45 min and the gradient program was as follows: 0–10 min (10–20% B), 10–16 min (20–40% B), 16–20 min (40–50% B), 20–25 min (50–70% B), 25–30 min (70% B), 30–40 min (70–10% B), 40–45 min (10% B). The column chamber temperature was set to 30 °C and the chromatograms were recorded at 275 nm. The DAD response was linear for all phenolics within the calibration ranges of 0.05–120 µg/mL with the correlation coefficients exceeding 0.9988. The quantification of analytes was based on the standard curves of the corresponding phenolics at a wavelength of 275 nm with the peak section used for the calculations. Individual phenolics were identified using their retention times and the method of standard addition.

### 2.7. Determination of Total Anthocyanin Content

Anthocyanins were extracted according to [40] and pH differential absorbance method was applied with a slight modification to evaluate total anthocyanin content [2]. First, ground nutraceutical mixture samples of 4 g was extracted in a mixture of 10 mL of methanol and 1 M HCl in the ratio of 85:15. After being extracted in a water bath (1 h at 35 °C) with shaking, the extract was centrifuged at 12,300× *g* for 15 min. 0.5 mL of the extract was then simultaneously diluted with 2.5 mL of 0.025 mol/L KCl (pH 1.0) and 0.4 mol/L CH_3_COONa·3H_2_O (pH 4.5). Finally, the absorbance was recorded at 510 nm and 700 nm by Lambda 25 (PerkinElmer, Waltham, MA, USA). Total monomeric anthocyanin content (TAC) was calculated as follows:

A = [(A_510_ − A_700_)_pH 1.0_ − (A_510_ − A_700_)_pH 4.5_] (1)

TAC (mg/L) = (A × MW × DF × 1000)/(ε × l)(2)

where: A is the absorbance of diluted sample, MW is the molecular weight for cyanidin-3-glucoside (449.2 g/mol), DF is the dilution factor, 1000 expresses the conversion from g to mg, ε is the molar absorptivity coefficient for cyanidin-3-glucoside (26.900 L/mol/cm), l is the length of the path in cm. The results were expressed as mg of cyanidin-3-glucoside equivalents per kg in dry weight (mg C3G/kg).

### 2.8. Determination of Individual Anthocyanin and Anthocyanidin Contents Using HPLC

The profile and quantity of selected anthocyanins and anthocyanidins were analyzed using the HPLC system Dionex UltiMate 3000 (Thermo Scientific, Waltham, MA, USA) with a diode-array detector (DAD). The analytes were separated by YMC-Triart C18 (150 × 3.0 mm; 5 μm) column (Waters, Milford, MA, USA). The injection volume was 20 μL, flow rate 1 mL/min, column temperature 25 °C and the analysis time was 35 min. As a solvent A, a mixture of ultrapure water/85% formic acid in a ratio of 90:10 was introduced into the column with solvent B consisting of acetonitrile. The mobile phase gradient was established as follows: 0–5 min (2% B), 5–20 min (5–25% B), 20–30 min (25–2% B), 30–35 min (2% B). The chromatograms were recorded at 520 nm [41]. Individual anthocyanins and anthocyanidins were identified by comparing their retention times to those of standards and evaluated by the method of standard addition. Their concentrations were calculated using linear regression equations based on the dependence of the peak areas on the concentrations of the analyte standards (0.05–80 µg/mL) with the correlation coefficients exceeding 0.9980. Data signals were processed with Chromeleon^TM^ 7.2 software (Dionex, Waltham, MA, USA).

### 2.9. Determination of Antioxidant Activity Values Using ABTS and DPPH Radicals

The final extracts of free, soluble bound and insoluble bound phenolic fractions prepared from nutraceutical cereal mixtures (Section 2.4) were used in the antioxidant activity assay based on deactivating ABTS and DPPH radicals. First, 7 mol/L ABTS stock solution was prepared by mixing with 60 mmol/L K_2_S_2_O_8_ in a volume ratio of 1:50. Then, it was incubated for 16 h at room temperature without sunlight. To prepare a working solution of ABTS radicals, 2.5 mL of stock solution was mixed with 97.5 mL of acetic buffer (pH 4.3). The sample extracts (100–200 µL) were then mixed with 24 mL of ABTS working solution. The decline of the absorbance was evaluated spectrophotometrically (Lambda 25, Perkin Elmer, Waltham, MA, USA) at 734 nm after the incubation at room temperature for 30 min [42]. Furthermore, the antioxidant activity values of the samples were measured using a DPPH radical scavenging activity assay [43]. Briefly, 450 µL of the phenolic extract were mixed with 8 mL of 0.17 M methanolic solution of DPPH radicals. The absorbance was monitored at 515 nm after being allowed to rest for 1 h without sunlight. Trolox in the concentrations of approximately 0 to 0.24 mmol/L was used as a reference standard in both antioxidant assays. The results of the determination of the antioxidant activity values were expressed in mmol or mol of Trolox equivalent per gram in dry basis (mmol or mol TE/g), respectively.

### 2.10. Determination of the Antioxidant Capacity of Water and Lipid-Soluble Compounds

The antioxidant capacities of water-soluble (ACW) and lipid-soluble (ACL) compounds of nutraceutical mixtures were established by photochemiluminiscence assays (PCL) combining a photochemical generation of superoxide anion radicals with a sensitive detection by chemiluminescence. Measurements were performed according to the study by [44] using ACL and ACW kits supplied by Analytik Jena AG (Jena, Germany). A Photochem^®^ biochemistry analyzer (Analytik Jena AG, Jena, Germany) was used in compliance with the instructions of the ACW and ACL protocols. The antioxidant capacity values of both water (ACW) and lipid-soluble (ACL) compounds of the nutraceutical mixtures were calculated using the calibration curve with Trolox as a standard. The results of ACW and ACL values were expressed as mmol of Trolox equivalent per kg of dry matter (mmol TE/kg). In addition, these results are also presented as the internal antioxidant capacity (IAC) quantifying the potential synergistic capacity of the sample. The IAC value is defined by the sum of ACW and ACL capacities of water and methanolic extracts of the nutraceutical mixtures with edible flowers. Five hundred µL of reagent 1 (Kit ACL) were added to a vial with Trolox (reagent 4, Kit ACL) and were stirred for 10–20 s to obtain the stock solution. Subsequently, it was diluted with reagent 1 so that 10 µL of this solution contained 1 nmol Trolox as a standard. Measurements were repeated four times. Similarly, 490 µL of reagent 1 (Kit ACW) and 10 µL of H_2_SO_4_ were added to the vial containing Trolox (reagent 4, Kit ACL) and stirred for 30 s. The resulting stock solution was diluted with reagent 1 to obtain the work solution whose volume of 10 μL contained 1 nmol Trolox as a calibration standard. The measurements were repeated four times. The exact amount of nutraceutical mixture (0.5 g) was accurately suspended in 5 mL of ultrapure water (for ACW value) or methanol (for ACL value) to be sonicated for 15 min. The sample was then centrifuged (Velocity 13μ; Dynamica Scientific Ltd., Newport Pargnell, UK) at 12,300× *g* for 10 min and afterwards, the supernatant was immediately diluted with reagent 1 of ACW or ACL kit (Analytik Jena, Jena, Germany).

### 2.11. Statistical Analysis

All analyses were repeated 5 or 8 times and their results were reported as mean ± standard deviation of dry weight. The Dean-Dixon test and one-way analysis of variance (ANOVA) were applied. Thereafter, Tukey’s test was used to identify differences between the means. The significance level was set to 5% (*p* < 0.05). Unistat 6.0 software (Unistat Ltd., London, UK) was used for the statistical evaluation.

## 3. Results and Discussion

### 3.1. Basic Chemical Parameters

The results of the basic chemical parameters measured as a part of the storage experiment under defined conditions are presented in Table S1. The amount of dry matter in the nutraceutical mixtures at the beginning of the experiment ranged between 90.5 and 91.5%. The regulation [12] specifies that the maximum moisture content for the flakes is 14%, however, the moisture content for cereal mixtures is not legislatively defined. The importance of monitoring moisture content is to prevent the occurrence of fungi that could become potential producers of mycotoxins, especially within the increasing humidity. Moreover, Maillard reactions, fatty acids oxidation and different enzymatic reactions can be observed. The ash content, a rough indicator of the sum of minerals, ranged from 2.16 to 2.55%. Compared to commercial mixtures with fruits, new nutraceutical mixtures included higher ash content [42]. Cereal flakes, especially gluten-free flakes, containing the remains of coating layers with deposits of mineral substances certainly contributed to that higher ash content. In addition, nuts, seeds, fruits, and edible flowers are also ingredients recognized as valuable foods with significant ash content [45,46,47].

### 3.2. The Evaluation of Total Phenolic and Anthocyanin Contents

At the beginning of the storage experiment (Table 2), the highest TPC value was observed in the gluten-free sample M4S (2430 mg GAE/kg) contrasting to the lowest TPC value assessed in the sample M1S (1170 mg GAE/kg). As shown in Table 2, high polyphenol concentrations were determined in free and soluble bound fractions. A significant variety of ingredients in the nutraceutical mixtures complicates the results commentary and the linking of specific data to the relevant substance employed in the recipe. Even though TPC contents in individual ingredients depend on various factors, and thus will often differ between each batch of products, the trend in their individual phenolic fractions content is likely to be maintained. The gluten-free M4S nutraceutical mixture contains ingredients with biologically active substances, namely fruits (blueberries, raspberries, barberries), roses, cornflowers, and black and red rice flakes. In particular, red and black rice flakes are significant providers of free polyphenols [48]. Considerable TPC concentrations are also typical for nuts, berry fruits, and edible flowers [49,50,51,52]. Providing edible flowers and herbs are processed gently (e.g., by drying), polyphenolic substances become concentrated and may reach up to two orders of magnitude higher concentrations (referred to dry weight of the sample) [18,23]. Therefore, they may notably affect TPC values despite their lower weight share in the mixtures. Hence the M4S gluten-free nutraceutical mixture may be recommended for the celiac diet. Soluble and insoluble bound phenolics can be released in the gastrointestinal tract (GIT) under specific conditions (acidic or alkaline pH) and during the fermentation by colonic bacteria and/or after the absorption by the intestinal wall. Their availability requires the activity of enzymes and microflora in the intestinal tissue [20,53].

As can be seen in Table 2, total anthocyanin content (TAC) in the nutraceutical mixtures varied between 322 and 663 mg C3G/kg. It can be supposed that the main sources of anthocyanins include blueberries, strawberries, barberries [54], and to a lesser extent also edible flowers, such as rose and mallow [55]. However, the bioavailability of anthocyanins from fruits can be significantly limited by their poor stability with an increasing pH [56,57]. Benvenuti et al. [55] reported a notable antioxidant activity caused by a high content of anthocyanins, especially in more pigmented flowers. Particularly coating layers of red wheat are emphasized considering their valuable anthocyanin contents [58].

### 3.3. The Effect of Storage Conditions on TPC a TAC Values

**Figure 2:**
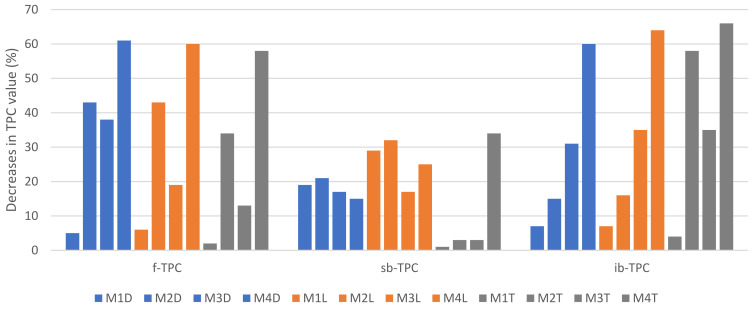
Percentage decreases in TPC concentrations for individual phenolic fractions of the M1–M4 nutraceutical mixtures under the defined storage conditions: f-TPC (free phenolics), sb-TPC (soluble bound phenolics), ib-TPC (insoluble bound phenolics), D (storage without sunlight, blue color), L (storage in the presence of sunlight, orange color), T (storage in thermostatic device, grey color).

The effect of the particular storage conditions on the concentrations of phenolics in the individual phenolic fractions are presented in Table 2 and the appropriate percentage reductions in their concentration values are depicted in Figure 2. Specifically, during the storage of the mixture M1L in the presence of daylight at a temperature of 23 °C free polyphenols decreased by 6% and they fell only by 2% when stored in a thermostatic device (M1T) at a temperature of 40 °C. The sample M1L demonstrated the highest decrease in polyphenols in soluble bound fractions (by 29%) in the presence of sunlight. The highest decrease of 43% in free phenolic contents was determined in the least stable sample during the storage in the presence of sunlight (M2L) and dark conditions (M2D) at laboratory temperature. Furthermore, the sample M2T showed the highest decrease in the concentrations of soluble bound phenolics (up to 58%) within the storage at 40 °C. The gluten-free samples M3 and M4 performed higher losses of polyphenols in individual fractions under the given storage conditions, in particular, the sample M4 appears to be the least stable in terms of polyphenol content (Figure 2).

**Figure 3:**
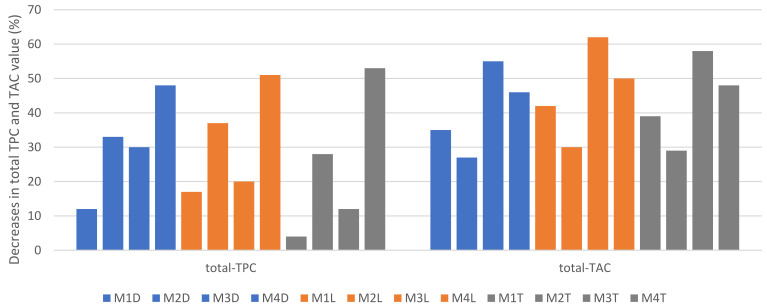
Percentage decreases in the concentrations of TPC and TAC values of the M1–M4 nutraceutical mixtures under the defined storage conditions: D (storage without sunlight, blue color), L (storage in the presence of sunlight, orange color), T (storage in thermostatic device, grey color).

The Influence of individual storage conditions on TPC and TAC in nutraceutical mixtures with edible flowers is presented in Figure 3 and Table 2. It could be summarized that the highest decreases in TPC were evaluated in the M4 nutraceutical mixture sample under all storage conditions. Polyphenolic substances were degraded by more than 48%. Even though the M4 mixture contains the highest value of phenolic substances, unfortunately, it also appears to be the least stable in terms of storage. The presence of rice flakes itself could have contributed to TPC degradation in this sample as the initiation of TPC degradation could have followed the grain hydrothermal treatment [10].

It may be assumed that hibiscus flower seems to contribute to the stability of the M1 nutraceutical mixture. It is acidic and creates a suitable stable environment, especially for anthocyanins [59]. It has been confirmed that fresh edible flowers with acid flavor had constant values of anthocyanins after 10-day cold storage at 4 °C [9]. If the mixtures were stored at 40 °C without access to sunlight, polyphenolic substances were generally more stable.

Concerning TAC (Figure 3, Table 2), all types of storage conditions provided a significant unfavorable effect on TAC values. Data shows that anthocyanin pigments were the most stable when stored at laboratory temperature in the absence of sunlight with their losses ranging from 27 to 55%. Furthermore, anthocyanin pigments of nutraceutical mixtures were less stable under the conditions with the access to sunlight than in a thermostatic device set to 40 °C. Gluten-free nutraceutical mixtures performed more losses in their TAC concentrations under all storage conditions. Anthocyanin stability is influenced by temperature and pH value [31]. At a temperature of 60 °C in the presence of sunlight, anthocyanin pigments degrade by more than 60–85% in only a few days, at room temperature they are stable for two weeks. In the presence of oxygen, they degrade even faster [60,61]. To maintain the quality of the product, this study employed the packaging material providing the air barrier to control the storage atmosphere and the samples were vacuumed during the packaging process, therefore, oxidation processes causing degradation of anthocyanin pigments should be negligible.

### 3.4. The Evaluation of Individual Flavonoids and Phenolic Acids by HPLC

Contents of individual phenolics in free, soluble, and insoluble bound fractions of the tested nutraceutical mixtures are depicted in Tables S2–S9. Protocatechuic and neochlorogenic acids were the main phenolic acids in free fraction of mixtures with edible flowers. Considering flavonoids, epicatechin and rutin were observed as the main free constituents in the mixtures with edible flowers (Tables S2 and S3). Regarding the soluble bound fraction (Tables S4 and S5), the main constituents of the nutraceutical mixtures presented epicatechin, catechin, rutin, and protocatechuic acid in all mixtures. Considering phenolics in insoluble bound fraction (Tables S6 and S7), significant amount of epicatechin and neochlorogenic acid were monitored in all mixtures. Moreover, the M3S and M4S gluten-free nutraceutical mixtures contained notable amounts of catechin and protocatechuic acid, respectively. In summary, the M1S and M2S nutraceutical mixtures with edible flowers (Tables S8 and S9) performed considerable concentrations of epicatechin (400 and 331 mg/kg), protocatechuic acid (220 and 233 mg/kg), neochlorogenic acid (103 and 87.3 mg/kg), catechin (97.1 and 99.1 mg/kg), and rutin (18.2 and 109 mg/kg), respectively. Similarly, gluten free nutraceutical mixtures contained high values of epicatechin (254 and 169 mg/kg), protocatechuic acid (208 and 185 mg/kg), catechin (195 and 130 mg/kg), rutin (147 and 110 mg/kg), neochlorogenic acid (84.6 and 118 mg/kg), and sinapic acid (38.0 and 40.4 mg/kg), respectively.

The variety of the nutraceutical mixtures complicates relating the specific data to the relevant ingredient used in the mixture recipe. For example, purple wheat, red and black rice serve as a rich source of protocatechuic acid [62,63], one of the most frequent phenolic acids in the nutraceutical mixtures. What is more, protocatechuic acid could also be provided by nuts, specifically almonds [64,65]. It is a general fact that pigmented kinds of berry fruits are rich in phenolics comprising a significant mass proportion of nutraceutical mixtures. Blueberries, strawberries, raspberries, and apples are especially excellent sources of protocatechuic acid [66,67]. In concern with rutin and quercetin, they are found in apples and barberries [68,69]. Edible flowers are another valuable source of polyphenolic substances [52,70], mainly represented by polyphenolic acids with generally predominant gallic and sinapic acids [70]; or gallic acid found in hibiscus flower [52].

### 3.5. The Effect of Storage Conditions on Individual Phenolic Acid and Flavonoid Contents

**Figure 4:**
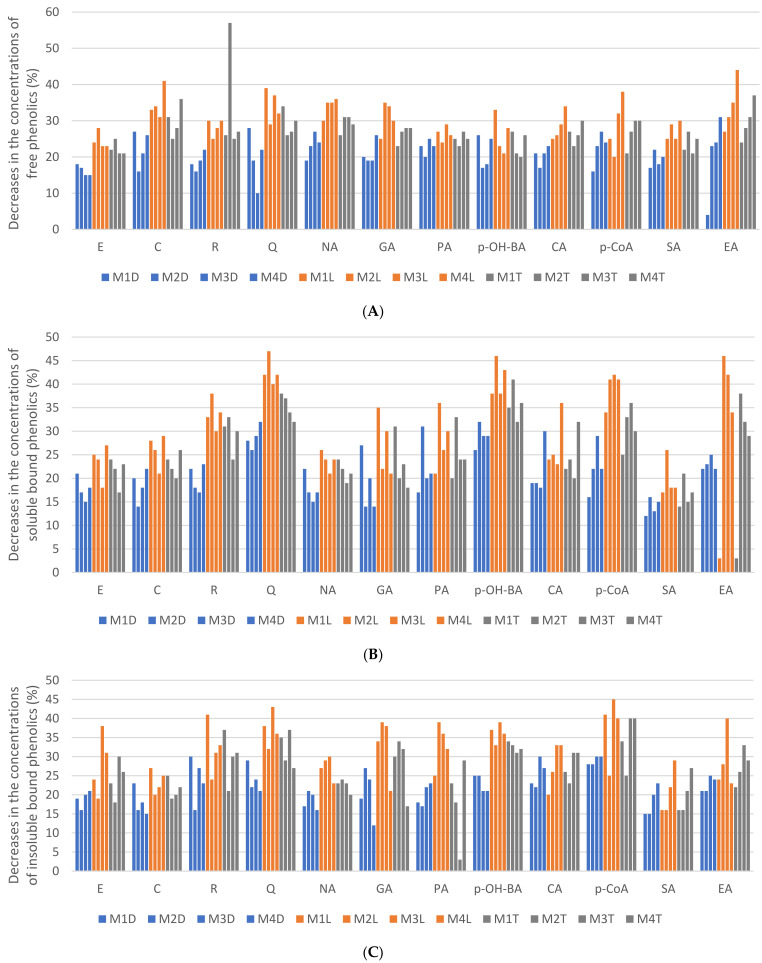

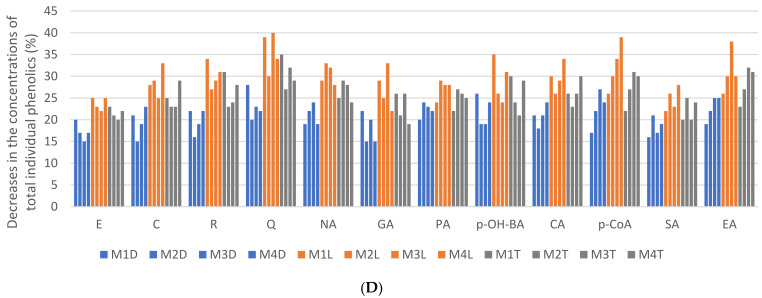
(**A**) Percentage decreases in the concentrations of free phenolics of the M1–M4 nutraceutical mixtures under the defined storage conditions: E (epicatechin), C (catechin), R (rutin), Q (quercetin), NA (neochlorogenic acid), GA (gallic acid), PA (protocatechuic acid), *p-*OH-BA- (*p-*hydroxybenzoic acid), CA (caffeic acid), *p-*CoA (*p-*coumaric acid), SA (sinapic acid), EA (ellagic acid), D (storage without sunlight, blue color), L (storage in the presence of sunlight, orange color), T (storage in thermostatic device, grey color). (**B**) Percentage decreases in the concentrations of soluble bound phenolics of the M1–M4 nutraceutical mixtures under the defined storage conditions: E (epicatechin), C (catechin), R (rutin), Q (quercetin), NA (neochlorogenic acid), GA (gallic acid), PA (protocatechuic acid), *p-*OH-BA- (*p-*hydroxybenzoic acid), CA (caffeic acid), *p-*CoA (*p-*coumaric acid), SA (sinapic acid), EA (ellagic acid), D (storage without sunlight, blue color), L (storage in the presence of sunlight, orange color), T (storage in thermostatic device, grey color). (**C**) Percentage decreases in the concentrations of insoluble bound phenolics of the M1–M4 nutraceutical mixtures under the defined storage conditions: E (epicatechin), C (catechin), R (rutin), Q (quercetin), NA (neochlorogenic acid), GA (gallic acid), PA (protocatechuic acid), *p-*OH-BA- (*p-*hydroxybenzoic acid), CA (caffeic acid), *p-*CoA (*p-*coumaric acid), SA (sinapic acid), EA (ellagic acid), D (storage without sunlight, blue color), L (storage in the presence of sunlight, orange color), T (storage in thermostatic device, grey color). (**D**) Percentage decreases in the concentrations of total phenolics of the M1–M4 nutraceutical mixtures under the defined storage conditions: E (epicatechin), C (catechin), R (rutin), Q (quercetin), NA (neochlorogenic acid), GA (gallic acid), PA (protocatechuic acid), *p-*OH-BA- (*p-*hydroxybenzoic acid), CA (caffeic acid), *p-*CoA (*p-*coumaric acid), SA (sinapic acid), EA (ellagic acid), D (storage without sunlight, blue color), L (storage in the presence of sunlight, orange color), T (storage in thermostatic device, grey color).

The effect of storage conditions on the contents of individual phenolic acids and flavonoids is shown in Tables S2–S9 with the appropriate percentage declines of individual phenolics in the phenolic fractions after the storage experiment summarized in Figure 4A–D.

Concerning individual free phenolics, their concentrations decreased more after the storage in the presence of daylight (approx. 23–44%) when compared to the storage in a thermostatic device (20–37%). The only exception is rutin in the M2T nutraceutical mixture, which degraded by 57% after the storage in a thermostatic device at 40 °C. When the nutraceutical mixtures were stored without the presence of sunlight, the percentage value of the degradation ranged from 4 to 31%. As could be seen in Figure 4A, ellagic acid, catechin, quercetin, neochlorogenic acid and, in gluten-free mixtures, *p-*coumaric acid degraded the most in the presence of sunlight. Epicatechin appeared to be the most stable phenolic which is in compliance with the study [52].

In regard tophenolics in the soluble bound fraction, the reductions in phenolic acids and flavonoids are displayed in Figure 4B. The highest decreases of their concentrations between 3–47% were measured when the mixtures were stored in the presence of sunlight at 23 °C. Specifically, quercetin degraded the most, followed by *p-*hydroxybenzoic, and *p-*coumaric acids specifically in the mixtures M2L–M4L. When stored in a thermostatic device, the decreases reached 41% with quercetin, *p-*hydroxybenzoic, and *p-*coumaric acids degrading the most significantly.

The highest decreases in individual phenolics in insoluble bound fractions were obtained while stored in the presence of sunlight with the values declining in the range from 16 to 45% (Figure 4C). Insoluble bound phenolics were the most stable when the mixtures were stored without the presence of sunlight with the level of degradation between 15–30%. Sinapic, protocatechuic, gallic and neochlorogenic acids, catechin and epicatechin seemed to be the most stable compounds.

From the overview of total losses of individual polyphenolic substances (Figure 4D) it is obvious that the presence of sunlight contributes to their highest degradation, followed by losses during the storage in a thermostatic device at 40 °C. Polyphenolic substances were the most stable when stored in the dark at 23 °C. In detail, quercetin, *p-*coumaric acid, ellagic acid, *p-*hydroxybenzoic acid, and rutin were the least stable phenolics when exposed to sunlight. In contrast, gallic, sinapic, and caffeic acids, epicatechin, and catechin were the most stable phenolic compounds when stored without the presence of sunlight. There are studies suggesting a low stability of caffeic acid in edible flowers and fruits [52,67,68]. However, if the mixtures are stored in the absence of sunlight, caffeic acid is monitored to be more stable.

Individual phenolics are synthesized by plants as secondary metabolites. Therefore, their concentrations are influenced by many factors including the plant species and its variety, maturity, as well as climatic and stress conditions and UV radiation. Zhou et al. [71] reported that during the storage period up to 6 months at the temperatures between 4 and 37 °C, gallic and caffeic acids decreased in bound phenolic fractions of brown rice. Another study showed a decline in phenolic compounds in blueberry during the storage at different temperatures reporting a reduction in the concentrations of gallic, caffeic, *p*-coumaric, and ellagic acids, catechin, and quercetin during the storage [72].

### 3.6. The Evaluation of Individual Anthocyanins and Anthocyanidins by HPLC

The records of individual anthocyanin and anthocyanidin contents are shown in Table 3 and Table 4. Delphinidin-3-glucoside, cyanidin-3-glucoside, and cyanidin-3-rutinoside were identified as the main anthocyanins. Delphinidin-3-glucoside showed its highest content of 69.4 mg/kg in the M2S nutraceutical mixture, followed by 47.6 mg/kg in the M4S nutraceutical mixture. This may stem from the presence of blueberries, strawberries, rose petals, blue cornflower, and mallow, all of them rich in delphinidin derivatives [41,73,74]. Peonidin presented with the lowest concentrations of less than 0.10 mg/kg, especially in the gluten-free nutraceutical mixtures. In accordance with the available published data, fruits, edible flowers, and pigmented flakes appear to be the most valuable providers of anthocyanins and anthocyanidins [41,74,75,76]. Blueberries are an excellent source of anthocyanin pigments, such as delphinidin, cyanidin, peonidin, and malvidin [41,75]. According to the report by [76], the most widespread anthocyanin in cherry is cyanidin-3-rutinoside followed by cyanidin-3-glucoside. A recent study by [73] showed that mallow is a good source of delphinidin-3-glucoside and cyanidin-3-glucoside, cyanidin-3-rutinoside was determined to be the main contributor to the content of anthocyanins in red wheat [77].

### 3.7. The Effect of Storage Conditions on Individual Anthocyanins and Anthocyanidins

**Figure 5:**
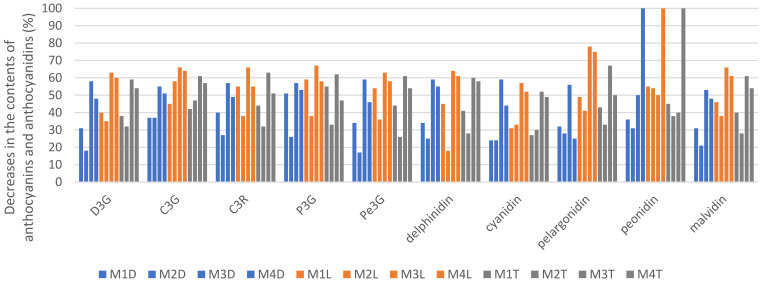
Percentage decreases in the contents of anthocyanins and anthocyanidins of the M1–M4 nutraceutical mixtures under the defined storage conditions: D3G (delphinidin-3-glucoside), C3G (cyanidin-3-glucoside), C3R (cyanidin-3-rutinoside), P3G (pelargonidin-3-glucoside), Pe3G (peonidin-3-glucoside), D (storage without sunlight, blue color), L (storage in the presence of sunlight, orange color), T (storage in thermostatic device, grey color).

Individual anthocyanin and anthocyanidin contents established in the nutraceutical mixtures tested under the specific storage conditions are presented in Table 3 and Table 4. Additionally, the appropriate percentage declines of their concentrations are shown in Figure 5. As can be seen, declines of anthocyanidin concentrations varied from 17 to 58% during the storage without the presence of sunlight at laboratory temperature and those of anthocyanidins ranged from 21 to 59%. The conditions with the presence of sunlight caused higher percentage decreases in concentrations when compared to the storage without sunlight. Here, the concentrations of glycosylated anthocyanins decreased by 35–67% and those of anthocyanidins by up to 78%. Interestingly, peonidin was not detected in the M4 gluten-free nutraceutical mixtures under any storage conditions or its concentrations were below its LOQ value. Peonidin may possibly degrade completely after a 1-year storage. The second least stable anthocyanidin was pelargonidin. These results confirmed that glycosylated forms of anthocyanins demonstrated a greater stability and individual anthocyanins in gluten-free mixtures had a lower stability than they had in the samples containing gluten. It is evident that anthocyanin pigments were the most stable when stored in the dark at room temperature (this storage condition applies especially to the nutraceutical mixtures containing gluten). After the storage in the dark, no decline in concentrations bigger than 60% was observed in any of monitored anthocyanins. In contrast, during the storage in the presence of sunlight, decreases of more than 60% were recorded in eight out of ten individual pigments mainly in the gluten-free mixtures.

A limited number of studies examining the stability of anthocyanins and anthocyanidins in gluten-free cereals complicates the commentary of the differences in their stability between gluten-free samples and samples containing gluten. Nevertheless, the content of pelargonidin-3-glucoside in apples decreased by 92% when stored at 35 °C during a 14-day period and the content of cyanidin-3-glucoside was measured below its LOQ limit [61]. Rubinskiene et al. [78] reported a decline in the concentrations of cyanidin-3-glucoside and cyanidin-3-rutinoside in blackcurrant during the storage at a temperature of 95 °C within 4 h. Zhang et al. [74] described the reduction in the stability of anthocyanins in mallow flowers in the presence of sunlight and explained that the stability of anthocyanins in flowers is supported by acidic conditions. It may be assumed from our results that the vacuum packaging of nutraceutical mixtures positively contributed to lower percentage losses in their contents with a possible prevention of the oxidation processes.

### 3.8. The Evaluation of Antioxidant Activity Assay

Antioxidant activity values of individual polyphenolic fractions in the nutraceutical mixtures determined using ABTS and DPPH radicals before and after the storage experiment are summarized in Table 5 and Table 6. At the start of the experiment, significantly high antioxidant activity values were measured in free phenolic fractions in the M2S, M3S and M4S nutraceutical mixtures. These results positively correlated with their high TPC values (see Tables S10–S13). Regarding total antioxidant activity values, varying results were obtained. Considering the method using ABTS radicals, the highest antioxidant value of 1780 mmol TE/kg was determined in the M2S nutraceutical mixture. Concerning the DPPH radical scavenging method, the highest antioxidant activity values were measured for the M2S and M4S samples with 15.3 mol TE/kg in both. These results confirm that nutraceutical cereal mixtures containing edible flowers offer a significant antioxidant potential. It may be assumed that the high antioxidant activity value of the M2S nutraceutical mixture was stimulated by adding fruits and edible flowers, specifically blueberries, strawberries, barberries, rose, and mallow.

In the assay using ABTS radicals, K_2_S_2_O_8_ oxidizes ABTS to its radical cation ABTS^•+^ which is intensely colored and antioxidant activity is measured as the ability of phenolic extracts to decrease its color [79]. DPPH^•^ radical is one of a few stable organic nitrogen radicals. The assay employing this radical is based on monitoring the reduction of the ability of antioxidants toward DPPH^•^ and the results are evaluated by measuring the decrease in its absorbance. DPPH color may be lost via either radical reaction or reduction as well as unrelated reactions with steric accessibility as a major determinant of the reaction [79].

The resulting value of antioxidant activity of the specific ingredient and thus of the mixtures can be influenced by many factors, including the climatic conditions, variety, genotypes, application of fertilizers during the cultivation, harvesting, and gentle technological processing [80]. Furthermore, the antioxidant activity values in the appropriate phenolic fractions of cereal grains are affected by the presence of color pigments in the coating layers [62]. Therefore, the flakes prepared from grains with colored coating layers were selected to be used in this study. Nuts and seeds belong to the ingredients possessing significant values of antioxidant activity as well [49]. Nevertheless, fruits and edible flowers are ingredients with one of the most significant antioxidant activity values [52,81]. Among many edible flowers, rose petals excel with their high values of TPC, TAC, and antioxidant activity, and they may be the reason for significantly higher antioxidant activities in the nutraceutical mixtures [9]. Interestingly, the antioxidant activity of some edible flowers is even higher than that of many blueberry varieties which are reported as one of the most powerful antioxidants [18].

The PCL assay involves the photochemical generation of superoxide O_2_^•–^ free radicals combined with a sensitive detection by chemiluminescence. Free radicals are detected with a luminol reagent, which acts as a photosensitizer as well as an oxygen radical detection reagent. In contrast to other commonly used antioxidant assays, this method is not restricted to a specific pH value or temperature range [79].

Considering the PCL method, ACW values of the water-soluble antioxidants were recorded between 50.1–84.3 mmol TE/g at the beginning of the storage experiment and ACL values of the lipid-soluble antioxidants varying from 25.4 to 43.9 mmol TE/g (see Table 7). The results obtained by the chemiluminescence assay emphasize that water-soluble biologically active compounds (ACW) show higher values of antioxidant activity compared to lipid-soluble antioxidants (ACL). Total IAC values of nutraceutical mixtures with edible flowers reached the values from 77.0 to 124 mmol TE/g. The highest IAC value was determined in the M2S nutraceutical mixture.

Current research in the food industry has experienced a growing interest in the measurement of PCL antioxidant activity due to the presence of antioxidants that are either water or lipid soluble. Above that, some of the antioxidants perform their own biological effect either in the hydrophilic or lipophilic body compartments [44]. The photochemical assay, in contrast to DPPH and ABTS, is considered to be one of the most biologically relevant assays. The antioxidant capacity of this in vitro method may reflect in vivo actions more closely [79]. It is commonly known that higher ACW values in fruits, teas, and herbs are usually attributed to high amounts of catechins, anthocyanins, phenolic and ascorbic acids; while carotenoids, chlorophylls, and tocopherols are considered as sources of antioxidant activity in lipid-soluble fractions (ACL) [8,82]. It is possible to estimate that the ingredients of nutraceutical mixtures rich in vitamin E, such as nuts and seeds, could contribute to antioxidant activity values of lipophilic fractions [82]. Similarly, Netzel et al. [8] have confirmed the linear relationship between ACW values and total phenolic content in fruit samples and herbs. Due to the variable composition of the nutraceutical mixtures, it may be assumed that fruits and edible flowers contribute significantly to the antioxidant activity expressed as ACW values followed by flakes with colored coating layers [8,15].

### 3.9. The Effect of Storage Conditions on Antioxidant Activity Assay

**Figure 6:**
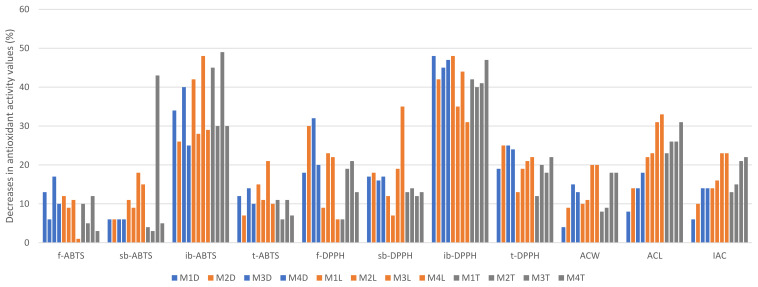
Percentage decreases in the antioxidant activity values for the individual phenolic fraction and the water- and lipid-soluble antioxidants of the M1–M4 nutraceutical mixtures under the defined storage conditions. Prefixes: f (free phenolic fraction), sb (soluble bound phenolic fraction), ib (insoluble bound phenolic fraction), t (total content), ABTS or DPPH (antioxidant activity measured by quenching ABTS or DPPH radicals), ACW (water soluble antioxidants), ACL (lipid-soluble antioxidants), IAC (integral antioxidant capacity), D (storage without sunlight, blue color), L (storage in the presence of sunlight, orange color), T (storage in thermostatic device, grey color).

Table 5 and Table 6 show the influence of the storage conditions on the antioxidant activity values of individual phenolic fractions in the nutraceutical mixtures and Figure 6 presents the appropriate percentage declines in the antioxidant values measured by using ABTS and DPPH radicals. The most negative effect was observed during the storage in a thermostatic device set to 40 °C and in the presence of sunlight at 23 °C with the decreases in antioxidant activity ranging from 28 to 49%. Considering free and soluble bound phenolic fractions, the declines in antioxidant activity values were lower (1–18%). The results determined using the DPPH radical followed the same trend as when ABTS radical was applied.

It could be stated that the M3 gluten-free nutraceutical mixture showed slightly higher reductions (11–21%) in antioxidant values within the elimination of ABTS radicals when compared to the other samples. Furthermore, the storage conditions with the presence of sunlight at 23 °C reduced the antioxidant activity the most in all nutraceutical mixtures (Figure 6). In the case of the DPPH method, the highest decreases in total antioxidant activity values were observed in both the M3 and M4 gluten-free samples varying between 18 and 25% under all types of the storage conditions. The storage without the presence of sunlight at a temperature of 23 °C performed a more negative effect on the antioxidant activity values in all samples.

Considering PCL measurements, the effect of storage conditions on ACW and ACL antioxidant capacity values is summarized in Table 7 and Figure 6. The results show that the highest decreases in ACW values, ranging from 10 to 20%, were observed during the storage in the presence of sunlight at 23 °C. The water-soluble antioxidants were the most stable when stored without access to sunlight and at laboratory temperature. Various results were obtained considering lipophilic fractions. The M1T and M2T nutraceutical mixtures performed the worst ACL values of 23 and 26%, respectively, when stored in a thermostatic device set to 40 °C. The gluten-free M3L and M4L nutraceutical mixtures presented the lowest ACL values of 31 and 33%, respectively, in the presence of sunlight at laboratory temperature. It is evident that fat-soluble as well as water-soluble antioxidant compounds are more stable when stored at laboratory temperature without access to sunlight. It could be summarized that the most significant effect on the value of the IAC was observed within the presence of sunlight at 23 °C, immediately followed by the storage in a thermostatic device at 40 °C. Considering the stability by evaluating the decrease in ACW, ACL, and IAC values, the M1S mixture appears to be the most stable followed by the sample M2S. The gluten-free mixtures were confirmed to be less stable irrespective of the used method.

It may be emphasized that the percentage decreases in the values of total antioxidant activities were lower than the percentage decreases in the content of total polyphenols and anthocyanins. This may be explained by the presence of other antioxidants outside the group of polyphenolic substances participating in the values of antioxidant activities. In addition, the values of antioxidant activities of plant extracts are related to the presence of certain individual phenolic compounds and their corresponding structures, with the positions and quantities of hydroxyl groups of a particular importance [81]. Determined results generally depend on the selected methodology, including the type of the extraction and solvent, reaction conditions within pH values, temperature, number of free radicals and the time provided for the reaction. For example, Klimczak et al. [83] reported decreases in antioxidant activity values in orange juice during the storage by 50 to 80% after only six months at a temperature of 38 °C without the presence of sunlight. In this study, total antioxidant activity values of the tested nutraceutical mixtures decreased significantly less which may stem from their better stability provided by the vacuum packing eliminating any oxidation processes. Monitored declines in antioxidant activity values have been reported to depend not only on the temperature and time of the storage of the fruit products, but also on their species and processing [29,81].

To prolong a shelf life in terms of the stability of antioxidant activity values and content of phenolic acids, flavonoids, especially anthocyanins, a dark, daylight-impermeable package and storage at room temperature can be recommended to potential manufacturers. Another important factor to consider is the vacuum packing of these nutraceutical mixtures or a protective atmosphere. What is more, antioxidants could be added to the cereal nutraceutical mixtures containing edible flowers to preserve their properties and extend their shelf life. Synthetic antioxidants, including highly effective butylhydroxyanisole in cereal mixtures and flakes [84], have become risky to apply due to their unknown possible harmful side effects. Therefore, ingredients and additives of natural origin are preferred as a safe solution with promising satisfactory results. Currently, tocopherols have been widely studied [84]. Moreover, additions of amino acids (phenylalanine, tyrosine, tryptophan) or polypeptide (*ε-*poly-L-lysine) are recommended to enhance the stability of anthocyanins during their storage. The most significant improvement has been observed after the application of tryptophan [60].

### 3.10. The Results of the Correlation Analysis

To identify and evaluate the main contributors to the antioxidant activity of each phenolic fraction, appropriate correlations between individual concentrations and antioxidant activities were calculated from total phenolic and anthocyanin contents, from contents of individual phenolic acids, flavonoids, anthocyanins, and anthocyanidins relating to the storage conditions. The corresponding Pearson´s correlation coefficients are displayed in Tables S10–S13.

Concerning the data obtained at the start of the storage experiment (Table S9), TPC and TAC contents of the nutraceutical mixtures positively correlated with the antioxidant activity determined using ABTS and DPPH radicals (*r* = 0.6207–0.8798) and with ACW and IAC values (*r* = 0.6599–0.8627). Correlations between total values of individual phenolics and antioxidant activity values obtained by ABTS and DPPH radicals proved delphinidin-3-glucoside (*r* = 0.9954), gallic acid, sinapic acid, cyanidin-3-rutinoside, *p-*hydroxybenzoic acid, delphinidin, malvidin, caffeic acid, peonidin, rutin and caffeic acid (*r* = 0.7294) as strong antioxidants. When discussing water-soluble antioxidants (ACW), the main contributors to the antioxidant capacity include cyanidin-3-rutinoside (*r* = 0.9222), gallic acid, sinapic acid, delphinidin-3-glucoside, malvidin, *p-*hydroxybenzoic acid, delphinidin, and caffeic acid (*r* = 0.5625). The results of the correlations between individual polyphenolic substances and IAC values confirm that the main contributors to the values of integral antioxidant capacity are delphinidin-3-glucoside (*r* = 0.9839), *p-*coumaric, gallic, sinapic and *p-*hydroxybenzoic acids, delphinidin, peonidin, and malvidin (*r* = 0.6538).

The influence of the storage conditions on the correlations between phenolic contents and antioxidant activity values is presented in Tables S11–S13. For the samples stored without the presence of sunlight, TPC and TAC values positively contributed to the antioxidant activity values (*r* = 0.3127–0.9958) (Table S11). Cyanidin-3-rutinoside (*r* = 0.9897) > delphinidin-3-glucoside > gallic acid > malvidin > *p-*hydroxybenzoic acid > sinapic acid > delphinidin and quercetin (*r* = 0.7643) contributed to the antioxidant activity values in this order. Furthermore, gallic acid, delphinidin-3-glucoside, cyanidin-3-rutinoside, delphinidin, and malvidin were confirmed as strong antioxidants in the case of PCL measurements. The storage conditions with the presence of sunlight (Table S12) showed the most negative effect on phenolic contents. The contributors to the antioxidant activity values were determined in the following order: gallic acid (*r* = 0.9839), cyanidin-3-rutinosid, delphinidin-3-glucoside, delphinidin, malvidin and sinapic acid (*r* = 0.7500). Gallic acid (*r* = 0.9790), cyanidin-3-rutinoside, and delphinidin-3-glucoside (*r* = 0.8815) were identified as strong antioxidants in water-soluble fractions (ACW), similarly, delphinidin-3-glucoside (*r* = 0.9284), malvidin, pelargonidin, delphinidin, cyanidin-3-rutinoside and quercetin (*r* = 0.8077) were strong antioxidants in lipid-soluble fractions (ACL) of the tested nutraceutical mixtures. Considering the storage conditions in a thermostatic device set to 40 °C (Table S13), cyanidin-3-rutinoside (*r* = 0.9775), gallic acid, delphinidin-3-glucoside, delphinidin, and sinapic acid (*r* = 0.8522) were recognized as the strongest contributors to the antioxidant activity values established by ABTS and DPPH radicals. It could be summarized that only anthocyanins and anthocyanidins, specifically delphinidin-3-glucoside (*r* = 0.9227), malvidin, delphinidin, cyanidin-3-rutinosid, and pelargonidin (*r* = 0.8749), were evaluated as significant contributors to ACL values. Concerning water-soluble antioxidants, gallic acid (*r* = 0.9763), cyanidin-3-rutinoside, delphinidin-3-glucoside, and delphinidin (*r* = 0.7618) significantly positively correlated with their ACW values.

It is generally known that different kinds of interactions between phenolics and free radicals depend on their chemical structure and specific antioxidant interactions in the tested systems. Even though theoretical correlations cannot fully reflect all metabolic pathways in the gastrointestinal tract and further factors, including the consumption of glycoside forms of phenolics, enzyme activity, or digestive factors influencing these interactions [85], they provide a valuable insight into this interesting issue.

## 4. Conclusions

This study has examined the concentrations and possible effects of storage conditions on the stability of individual phenolic acids, flavonols, flavones, flavanols, anthocyanins, anthocyanidins, and antioxidant activity values of nutraceutical mixtures with edible flowers. The measurement results showed that polyphenolic substances in nutraceutical mixtures occur mostly in the free faction followed by the soluble bound. The highest number of phenolic substances was recorded in the M4 mixture (containing mainly red and black rice flakes, rose, blue cornflower, raspberries, and blueberries; unfortunately, it also appears to be the least stable within all storage conditions. Gluten-free nutraceutical mixtures contained significantly higher total anthocyanin contents compared to the mixtures with flakes containing gluten. All samples showed high concentrations of epicatechin, protocatechuic acid, neochlorogenic acid, catechin, rutin, and sinapic acid; furthermore, delphinidin-3-glucoside, cyanidin-3-glucoside, cyanidin-3-rutinoside, and delphinidin were evaluated to be the main anthocyanin pigments. Concerning the storage experiment, gallic, sinapic, and caffeic acids, epicatechin, and catechin proved to be the most stable phenolics when the nutraceutical mixtures were stored without the presence of sunlight. In contrast, *p-*coumaric, ellagic, *p-*hydroxybenzoic acids, and rutin were the least stable substances when the samples were exposed to sunlight. Similarly, the concentrations of glycosylated anthocyanins decreased by 35–67% and those for anthocyanidins by up to 78% when stored in the presence of sunlight. Peonidin, pelargonidin and malvidin were identified to be the least stable anthocyanidins, contrasting to glycosylated forms of anthocyanins which demonstrated great stability. Regarding antioxidant activity measurements, the most considerable decreases in antioxidant activity values measured by the DPPH method under all types of the storage conditions were recorded in gluten-free samples. Concerning using ABTS radicals, the most negative effect on antioxidant values was evaluated for the storage in the presence of sunlight. The results obtained by the PCL assay showed that water-soluble biologically active compounds of nutraceutical mixtures (ACW) presented with higher antioxidant activities compared with lipid-soluble antioxidants (ACL). The decreases in ACW values between 10 and 20% were measured during the storage in the presence of sunlight were lower than in lipid-soluble antioxidant fraction for gluten-containing mixtures stored in a thermostatic device at 40 °C and stored in the presence of sunlight at laboratory temperature. TPC and TAC values of the nutraceutical mixtures positively correlated with the antioxidant activity measured by scavenging ABTS and DPPH radicals and with ACW and IAC values. Consequently, delphinidin-3-glucoside, gallic acid, sinapic acid, cyanidin-3-rutinoside, *p-*hydroxybenzoic acid, delphinidin, malvidin, caffeic acid, peonidin, rutin, and caffeic acid were evaluated as the main contributors to the antioxidant activity.

This unique study was not only focused on the determination of individual biologically active substances from the range of polyphenols, but also on their stability during storage. It is evident that nutraceutical mixtures are a great source of phenolic bioactive compounds with high performing antioxidant activities. The storage experiment has confirmed that the most significant detrimental effect on total and individual phenolics and anthocyanin pigments in all samples of the storage are those in the presence of sunlight at 23 °C followed by the storage in a thermostatic device set to 40 °C after one-year storage. In terms of the stability of individual phenolic substances, phenolics were the most stable when stored in the dark at 23 °C. These results have proved that gluten-free nutraceutical mixtures as the least stable under all types of the storage conditions when taking into account their antioxidant activity values, their decline in TPC and TAC values, and of course, their largest decreases in individual phenolic concentrations. Although gluten-free nutraceutical mixtures showed only a low stability, they still contain significant amounts of phenolic compounds responsible for their substantial antioxidant activity. Therefore, they could be newly proposed as suitable for patients suffering from celiac disease in order to enrich their diet. Taking this into future consideration, these mixtures should be packed in bags impervious to daylight. What is more, it was confirmed that using vacuum technology may contribute to the better stability of the contents of these valuable bioactive substances. Our study provides the field new perspectives for developing and involving dietary supplements with high stability of their biologically active compounds. Their stability should be enhanced even more by the addition of natural antioxidants in the future, which will be the subject of subsequent research.

## 5. Patents

Patent No. 306520, 2017. Mlček, J.; Sumczynski, D. Nutraceutical food mixture. Industrial Property Office of the Czech Republic, Prague, Czech Republic.

## Figures and Tables

**Table 1:** The specific composition of the nutraceutical mixtures with edible flowers.

Nutraceutical Mixtures Containing Gluten				Gluten-Free Nutraceutical Mixtures			
**M1S**	**g**	**M2S**	**g**	**M3S**	**g**	**M4S**	**g**
oat flakes	20	oat flakes	20	red rice flakes	20	red rice flakes	20
red wheat flakes	20	rye flakes	20	white teff flakes	20	black rice flakes	20
rye flakes	20	kamut flakes	20	black quinoa flakes	20	white quinoa flakes	20
hemp	2	hemp	2	hemp	2	hemp	2
almond	8	almond	8	almond	8	almond	8
hibiscus	2.5	rose	2	red cornflower	1.5	rose	1
lavender	0.5	mallow	1	blue cornflower	1.5	blue cornflower	2
raspberries	7	barberries	6	barberries	7	raspberries	7
barberries	6	apples	8	blueberries	7	barberries	6
apples	7	blueberries	8	cherries	7	apples	7
blueberries	7	strawberries	5	goldenberries	6	blueberries	7

Oat flakes (*Avena sativa*), red wheat flakes (*Triticum aestivum* var. *milturum*), rye flakes (*Secale cereale*), hibiscus (*Hibiscus sabdariffa*), lavender (*Lavandula angustifolia*), kamut flakes (*Triticum turgidum* subsp. *turanicum*), rose (*Rosa centifolia*), mallow (*Malva sylvestris* var. *mauritiana*), rice flakes (*Oryza sativa*), teff flakes (*Eragrostis tef*), quinoa flakes (*Chenopodium quinoa*), cornflower (*Centaurea cyanus*).

**Table 2:** Total phenolic and anthocyanin contents of nutraceutical mixtures under the defined storage conditions.

TPC	M1S	M1D	M1L	M1T	M2S	M2D	M2L	M2T
(mg GAE/kg)								
f-TPC	485 ± 10 ^a,A^	463 ± 10 ^b,A^	457 ± 10 ^b,A^	476 ± 10 ^c,A^	977 ± 20 ^a,B^	562 ± 10 ^b,B^	554 ± 10 ^b,B^	640 ± 10 ^c,B^
sb-TPC	560 ± 10 ^a,A^	455 ± 10 ^b,A^	400 ± 10 ^c,A^	554 ± 20 ^a,A^	453 ± 10 ^a,B^	356 ± 10 ^b,B^	308 ± 10 ^c,B^	441 ± 10 ^d,B^
ib-TPC	123 ± 10 ^a,A^	115 ± 5 ^b,A^	114 ± 8 ^b,A^	93 ± 5 ^c,A^	183 ± 10 ^a,B^	156 ± 2 ^b,B^	153 ± 10 ^b,B^	77 ± 10 ^c,B^
total-TPC	1170 ± 17 ^a,A^	1033 ± 15 ^b,A^	971 ± 16 ^c,A^	1123 ± 23 ^d,A^	1610 ± 25 ^a,B^	1074 ± 15 ^b,B^	1015 ± 17 ^c,B^	1158 ± 17 ^d,B^
	**M3S**	**M3D**	**M3L**	**M3T**	**M4S**	**M4D**	**M4L**	**M4T**
f-TPC	758 ± 20 ^a,C^	471 ± 20 ^b,A^	615 ± 10 ^c,C^	658 ± 20 ^d,C^	1470 ± 20 ^a,D^	566 ± 10 ^b,B^	586 ± 10 ^b,c,D^	615 ± 10 ^c,D^
sb-TPC	528 ± 10 ^a,C^	437 ± 10 ^b,C^	440 ± 10 ^b,C^	514 ± 10 ^c,C^	670 ± 10 ^a,D^	570 ± 20 ^b,D^	503 ± 20 ^c,D^	440 ± 10 ^d,B^
ib-TPC	144 ± 10 ^a,C^	100 ± 10 ^b,C^	96 ± 10 ^c,C^	93 ± 10 ^c,A^	294 ± 10 ^a,D^	120 ± 10 ^b,A^	105 ± 7 ^c,D^	99 ± 10 ^c,A^
total-TPC	1430 ± 25 ^a,C^	1008 ± 25 ^b,C^	1151 ± 17 ^c,C^	1265 ± 25 ^d,C^	2430 ± 25 ^a,D^	1256 ± 25 ^b,D^	1194 ± 23 ^c,D^	1154 ± 17 ^c,B^
**TAC**	**M1S**	**M1D**	**M1L**	**M1T**	**M2S**	**M2D**	**M2L**	**M2T**
(mg C3G/kg)								
	322 ± 2 ^a,A^	208 ± 1 ^b,A^	187 ± 2 ^c,A^	198 ± 1 ^d,A^	663 ± 2 ^a,B^	487 ± 2 ^b,B^	465 ± 1 ^c,B^	472 ± 2 ^d,B^
	**M3S**	**M3D**	**M3L**	**M3T**	**M4S**	**M4D**	**M4L**	**M4T**
	386 ± 2 ^a,C^	174 ± 5 ^b,C^	146 ± 5 ^c,C^	161 ± 4 ^d,C^	424 ± 2 ^a,D^	231 ± 2 ^b,d,D^	212 ± 4 ^c,d,D^	219 ± 2 ^d,D^

The results are presented as means in dry matter ± SD, n = 8 (the mean of eight measurements). For each variable and effect, values followed with different letters are significantly different at *p* < 0.05. M (appropriate nutraceutical mixture), S (start of storage experiment), D (storage condition without the presence of sunlight at 23 °C), L (storage condition in the presence of sunlight at 23 °C), T (storage in thermostatic device at 40 °C), TPC (total phenolic content), GAE (gallic acid equivalent), TAC (total anthocyanin content, C3G (cyanidin-3-glucoside equivalent), f-TPC (free phenolics), sb-TPC (soluble phenolics), ib-TPC (insoluble bound phenolics).

**Table 3:** Individual anthocyanin and anthocyanidin contents of the M1 and M2 nutraceutical mixtures under the defined storage conditions.

Anthocyanins (mg/kg)	M1S	M1D	M1L	M1T	M2S	M2D	M2L	M2T
D3G	5.35 ± 0.15 ^a,A^	3.68 ± 0.01 ^b,A^	3.21 ± 0.07 ^c,A^	3.34 ± 0.11 ^d,A^	69.4 ± 1.2 ^a,B^	51.8 ± 0.1 ^b,B^	45.1 ± 0.8 ^c,B^	47.2 ± 0.5 ^d,B^
C3G	43.8 ± 0.4 ^a,A^	27.8 ± 0.2 ^b,A^	23.9 ± 0.4 ^c,A^	25.4 ± 0.2 ^d,A^	0.19 ± 0.03 ^a,B^	0.12 ± 0.01 ^b,B^	0.08 ± 0.01 ^c,B^	0.10 ± 0.01 ^d,B^
C3R	2.34 ± 0.03 ^a,A^	1.41 ± 0.01 ^b,A^	1.05 ± 0.02 ^c,A^	1.32 ± 0.04 ^d,A^	32.1 ±1.2 ^a,B^	23.4 ± 0.1 ^b,B^	19.8 ± 0.2 ^c,B^	21.7 ± 1.0 ^d,B^
P3G	1.18 ± 0.02 ^a,A^	0.58 ± 0.01 ^b,A^	0.48 ± 0.01 ^c,A^	0.53 ± 0.01 ^d,A^	18.0 ± 0.5 ^a,B^	13.4 ± 0.01 ^b,B^	11.1 ± 0.5 ^c,B^	12.1 ± 0.7 ^d,B^
Pe3G	0.41 ± 0.04 ^a,A^	0.27 ± 0.04 ^b,A^	0.19 ± 0.01 ^c,A^	0.23 ± 0.02 ^d,A^	0.42 ± 0.05 ^a,A^	0.35 ± 0.01 ^b,B^	0.27 ± 0.04 ^c,B^	0.31 ± 0.01 ^d,B^
**Anthocyanidins** **(mg/kg)**	**M1S**	**M1D**	**M1L**	**M1T**	**M2S**	**M2D**	**M2L**	**M2T**
Delphinidin	1.04 ± 0.05 ^a,A^	0.69 ± 0.1 ^b,A^	0.57 ± 0.04 ^c,A^	0.61 ± 0.04 ^d,A^	38.2 ± 1.5 ^a,B^	28.6 ± 0.8 ^b,B^	24.9 ± 0.7 ^c,B^	27.4 ± 1.4 ^d,B^
Cyanidin	4.13 ± 0.21 ^a,A^	3.14 ± 0.12 ^b,A^	2.87 ± 0.12 ^c,A^	3.02 ± 0.11 ^d,A^	3.16 ± 0.12 ^a,B^	2.39 ± 0.02 ^b,B^	2.12 ± 0.02 ^c,B^	2.21 ± 0.10 ^c,B^
Pelargonidin	0.37 ± 0.02 ^a,A^	0.25 ± 0.02 ^b,A^	0.19 ± 0.01 ^c,A^	0.21 ± 0.01 ^d,A^	0.54 ± 0.03 ^a,B^	0.39 ± 0.01 ^b,B^	0.32 ± 0.01 ^c,B^	0.36 ± 0.03 ^d,B^
Peonidin	0.11 ± 0.01 ^a,A^	0.07 ± 0.01 ^b,A^	0.05 ± 0.01 ^c,A^	0.06 ± 0.01 ^c,A^	0.26 ± 0.01 ^a,B^	0.18 ± 0.01 ^b,B^	0.12 ± 0.02 ^c,B^	0.16 ± 0.01 ^d,B^
Malvidin	0.35 ± 0.02 ^a,A^	0.24 ± 0.01 ^b,A^	0.19 ± 0.01 ^c,A^	0.21 ± 0.02 ^d,A^	0.39 ± 0.02 ^a,B^	0.31 ± 0.01 ^b,B^	0.24 ± 0.01 ^c,B^	0.28 ± 0.02 ^d,B^

The results are presented as means in dry matter ± SD, n = 5 (the mean of five measurements). For each variable and effect, values followed with different letters are significantly different at *p* < 0.05. D3G (delphinidin-3-glucoside), C3G (cyanidin-3-glucoside), C3R (cyanidin-3-rutinoside), P3G (pelargonidin-3-glucoside), Pe3G (peonidin-3-glucoside), S (start of storage experiment), D (storage condition without the presence of sunlight at 23 °C), L (storage condition in the presence of sunlight at 23 °C), T (storage in thermostatic device at 40 °C).

**Table 4:** Individual anthocyanin and anthocyanidin contents of the M3 and M4 nutraceutical mixtures under the defined storage conditions.

Anthocyanins (mg/kg)	M3S	M3D	M3L	M3T	M4S	M4D	M4L	M4T
D3G	8.27 ± 0.32 ^a,A^	3.46 ± 0.02 ^b,A^	3.02 ± 0.21 ^c,A^	3.39 ± 0.12 ^b,A^	47.6 ± 1.3 ^a,B^	24.7 ± 0.9 ^b,B^	18.9 ± 0.3 ^c,B^	21.9 ± 0.5 ^d,B^
C3G	18.2 ± 0.3 ^a,A^	8.11 ± 0.02 ^b,A^	6.21 ± 0.44 ^c,A^	7.12 ± 0.2 ^d,A^	23.2 ± 1.1 ^a,B^	11.4 ± 0.09 ^b,B^	8.24 ± 0.1 ^c,B^	9.87 ± 1.7 ^d,B^
C3R	14.7 ± 0.14 ^a,A^	2.01 ± 0.01 ^b,A^	1.59 ± 0.10 ^c,A^	1.75 ± 0.11 ^d,A^	24.0 ±0.6 ^a,B^	12.3 ± 0.05 ^b,B^	10.7 ± 0.6 ^c,B^	11.7 ± 0.5 ^d,B^
P3G	17.9 ± 0.6 ^a,A^	7.63 ± 0.03 ^b,A^	5.98 ± 0.4 ^c,A^	6.74 ± 0.4 ^d,A^	0.19 ± 0.03 ^a,B^	0.09 ± 0.01 ^b,B^	0.08 ± 0.01 ^c,B^	0.10 ± 0.13 ^d,B^
Pe3G	8.77 ± 0.31 ^a,A^	3.62 ± 0.04 ^b,A^	3.21 ± 0.01 ^c,A^	3.41 ± 0.21 ^d,A^	1.60 ± 0.04 ^a,B^	0.87 ± 0.01 ^b,B^	0.67 ± 0.02 ^c,B^	0.74 ± 0.34 ^d,B^
**Anthocyanidins** **(mg/kg)**	**M3S**	**M3D**	**M3L**	**M3T**	**M4S**	**M4D**	**M4L**	**M4T**
Delphinidin	24.7 ± 1.5 ^a,A^	10.2 ± 1.4 ^b,A^	8.77 ± 0.08 ^c,^	9.78 ± 0.7 ^d,A^	19.4 ± 0.5 ^a,B^	8.78 ± 0.9 ^b,B^	7.65 ± 0.2 ^c,B^	8.24 ± 0.5 ^b,B^
Cyanidin	0.75 ± 0.05 ^a,A^	0.31 ± 0.02 ^b,A^	0.32 ± 0.04 ^b,A^	0.36 ± 0.01 ^c,A^	5.16 ± 0.20 ^a,B^	2.88 ± 0.10 ^b,B^	2.47 ± 0.10 ^c,B^	2.61 ± 0.02 ^d,B^
Pelargonidin	0.09 ± 0.01 ^a,A^	0.04 ± 0.01 ^b,d,A^	0.02 ± 0.01 ^c,d,A^	0.03 ± 0.01 ^d,A^	0.04 ± 0.01 ^a,B^	0.03 ± 0.01 ^b,d,A^	0.01 ± 0.01 ^c,d,A^	0.02 ± 0.01 ^d,A^
Peonidin	0.10 ± 0.01 ^a^	0.05 ± 0.01 ^b^	0.05 ± 0.01 ^b^	0.06 ± 0.01 ^b^	<0.01	ND	ND	ND
Malvidin	0.38 ± 0.03 ^a,A^	0.17 ± 0.02 ^b,A^	0.13 ± 0.01 ^c,A^	0.15 ± 0.01 ^d,A^	0.46 ± 0.04 ^a,B^	0.24 ± 0.04 ^b,B^	0.18 ± 0.01 ^c,B^	0.21 ± 0.01 ^d,B^

The results are presented as means in dry matter ± SD, n = 5 (the mean of five measurements). For each variable and effect, values followed with different letters are significantly different at *p* < 0.05. D3G (delphinidin-3-glucoside), C3G (cyanidin-3-glucoside), C3R (cyanidin-3-rutinoside), P3G (pelargonidin-3-glucoside), Pe3G (peonidin-3-glucoside), S (start of storage experiment), D (storage condition without the presence of sunlight at 23 °C), L (storage condition in the presence of sunlight at 23 °C), T (storage in thermostatic device at 40 °C). LOQ peonidin: 0.0001 mg/kg.

**Table 5:** Antioxidant activity values of nutraceutical mixtures under the defined storage conditions measured using ABTS radicals.

Antioxidant Activity (mmol TE/g)	M1S	M1D	M1L	M1T	M2S	M2D	M2L	M2T
f-ABTS	733 ± 20 ^a,A^	635 ± 10 ^b,A^	644 ± 10 ^c,A^	659 ± 10 ^d,A^	838 ± 20 ^a,B^	787 ± 10 ^b,B^	759 ± 20 ^c,B^	797 ± 20 ^b,B^
sb-ABTS	741 ± 20 ^a,A^	699 ± 10 ^b,A^	661 ± 10 ^c,A^	710 ± 10 ^d,A,C^	743 ± 10 ^a,A^	702 ± 10 ^b,A^	674 ± 10 ^c,B^	722 ± 20 ^d,B,C^
ib-ABTS	152 ± 20 ^a,A^	101 ± 5 ^b,A^	88 ± 10 ^c,A^	84 ± 5 ^c,A^	219 ± 10 ^a,B^	163 ± 10 ^b,B^	158 ± 10 ^b,c,B^	153 ± 10 ^c,B^
total-ABTS	1630 ± 40 ^a,A^	1440 ± 20 ^b,A^	1390 ± 20 ^c,A^	1450 ± 20 ^b,A^	1780 ± 30 ^a,B^	1650 ± 20 ^b,B^	1590 ± 30 ^c,B^	1670 ± 30 ^d,B^
	**M3S**	**M3D**	**M3L**	**M3T**	**M4S**	**M4D**	**M4L**	**M4T**
f-ABTS	772 ± 10 ^a,C^	639 ± 10 ^b,A^	627 ± 10 ^c,C^	681 ± 10 ^d,C^	810 ± 14 ^a,D^	731 ± 20 ^b,C^	799 ± 10 ^c,D^	788 ± 10 ^c,B^
sb-ABTS	742 ± 10 ^a,A^	700 ± 20 ^b,A^	608 ± 10 ^c,C^	718 ± 10 ^d,C^	700 ± 5 ^a,B^	657 ± 10 ^b,B^	597 ± 10 ^c,C^	644 ± 10 ^b,D^
ib-ABTS	136 ± 10 ^a,C^	82 ± 10 ^b,C^	71 ± 10 ^c,C^	69 ± 10 ^c,C^	232 ± 2 ^a,D^	175 ± 10 ^b,D^	164 ± 2 ^c,B^	162 ± 10 ^c,D^
total-ABTS	1650 ± 20 ^a,A^	1420 ± 30 ^b,A^	1310 ± 20 ^c,C^	1470 ± 20 ^d,A^	1740 ± 10 ^a,C^	1560 ± 30 ^b,C^	1560 ± 20 ^b,D^	1610 ± 20 ^c,C^

The results are presented as means in dry matter ± SD, n = 8 (the mean of eight measurements). For each variable and effect, values followed with different letters are significantly different at *p* < 0.05. TE (Trolox equivalent), ABTS (2,2-azinobis (3-ethylbenzo-thiazoline-6-sulfonic acid) diammonium salt), prefixes: f (free phenolic fraction), sb (soluble bound phenolic fraction), ib (insoluble bound phenolic fraction), S (start of storage experiment), D (storage condition without the presence of sunlight at 23 °C), L (storage condition in the presence of sunlight at 23 °C), T (storage in thermostatic device at 40 °C).

**Table 6:** Antioxidant activity values of nutraceutical mixtures under the defined storage conditions measured using DPPH radicals.

Antioxidant Activity (mmol TE/g)	M1S	M1D	M1L	M1T	M2S	M2D	M2L	M2T
f-DPPH	5.45 ± 0.03 ^a,A^	4.46 ± 0.01 ^b,A^	4.94 ± 0.02 ^c,A^	5.10 ± 0.02 ^d,A^	7.33 ± 0.03 ^a,B^	5.10 ± 0.02 ^b,B^	5.62 ± 0.02 ^c,B^	5.94 ± 0.02 ^d,B^
sb-DPPH	6.08 ± 0.02 ^a,A^	5.04 ± 0.01 ^b,A^	5.34 ± 0.02 ^c,A^	5.29 ± 0.01 ^d,A^	5.52 ± 0.01 ^a,B^	4.50 ± 0.01 ^b,B^	5.14 ± 0.01 ^c,B^	4.74 ± 0.02 ^d,B^
ib-DPPH	0.67 ± 0.01 ^a,A^	0.35 ± 0.02 ^b,A^	0.35 ± 0.02 ^b,A^	0.39 ± 0.01 ^c,A^	2.49 ± 0.04 ^a,B^	1.44 ± 0.02 ^b,B^	1.62 ± 0.02 ^c,B^	1.50 ± 0.01 ^b,B^
total-DPPH	12.2 ± 0.1 ^a,A^	9.85 ± 0.03 ^b,A^	10.6 ± 0.1 ^c,A^	10.8 ± 0.1 ^d,A^	15.3 ± 0.1 ^a,B^	11.0 ± 0.1 ^b,B^	12.4 ± 0.1 ^c,B^	12.2 ± 0.1 ^c,B^
	**M3S**	**M3D**	**M3L**	**M3T**	**M4S**	**M4D**	**M4L**	**M4T**
f-DPPH	6.82 ± 0.04 ^a,C^	4.63 ± 0.01 ^b,C^	5.35 ± 0.02 ^c,C^	5.41 ± 0.02 ^c,C^	6.64 ± 0.02 ^a,D^	5.28 ± 0.03 ^b,D^	6.27 ± 0.03 ^c,D^	5.40 ± 0.02 ^d,C^
sb-DPPH	6.44 ± 0.03 ^a,C^	5.42 ± 0.02 ^b,C^	5.24 ± 0.03 ^c,C^	5.64 ± 0.02 ^d,C^	6.06 ± 0.04 ^a,A^	5.02 ± 0.01 ^b,A^	3.92 ± 0.01 ^c,D^	5.26 ± 0.04 ^d,A^
ib-DPPH	0.68 ± 0.01 ^a,A^	0.37 ± 0.02 ^b,A^	0.38 ± 0.02 ^b,A^	0.40 ± 0.02 ^c,A^	2.60 ± 0.03 ^a,C^	1.39 ± 0.01 ^b,C^	1.80 ± 0.02 ^c,C^	1.37 ± 0.02 ^b,C^
total-DPPH	13.9 ± 0.1 ^a,C^	10.4 ± 0.1 ^b,C^	11.0 ± 0.1 ^c,C^	11.5 ± 0.1 ^d,C^	15.3 ± 0.1 ^a,B^	11.7 ± 0.1 ^b,D^	12.0 ± 0.1 ^c,D^	12.0 ± 0.1 ^c,B^

The results are presented as means in dry matter ± SD, n = 8 (the mean of eight measurements). For each variable and effect, values followed with different letters are significantly different at *p* < 0.05. TE (Trolox equivalent), DPPH (2,2-diphenyl-1-picrylhydrazyl), prefixes: f (free phenolic fraction), sb (soluble bound phenolic fraction), ib (insoluble bound phenolic fraction), S (start of storage experiment), D (storage condition without the presence of sunlight at 23 °C), L (storage condition in the presence of sunlight at 23 °C), T (storage in thermostatic device at 40 °C).

**Table 7:** Antioxidant activity values are determined in nutraceutical mixtures under the defined storage conditions measured using PCL.

Antioxidant Capacity	M1S	M1D	M1L	M1T	M2S	M2D	M2L	M2T
(mmol TE/kg)								
ACW	50.1 ± 1.0 ^a,A^	47.9 ± 0.4 ^b,A^	45.1 ± 0.5 ^c,A^	46.1 ± 0.4 ^d,A^	79.7 ± 0.7 ^a,B^	72.8 ± 0.4 ^b,B^	70.7 ± 0.5 ^c,B^	72.8 ± 0.5 ^d,B^
ACL	26.9 ± 0.4 ^a,A^	24.8 ± 0.4 ^b,A^	21.0 ± 0.4 ^c,A^	20.7 ± 0.5 ^c,A^	43.9 ± 1.0 ^a,B^	37.9 ± 0.4 ^b,B^	33.6 ± 0.3 ^c,B^	32.7 ± 0.2 ^d,B^
IAC	77.0 ± 1.0 ^a,A^	72.7 ± 0.5 ^b,A^	66.1 ± 0.6 ^c,A^	66.8 ± 0.6 ^c,A^	124 ± 1 ^a,B^	111 ± 1 ^b,B^	104 ± 1 ^c,B^	106 ± 1 ^c,B^
	**M3S**	**M3D**	**M3L**	**M3T**	**M4S**	**M4D**	**M4L**	**M4T**
ACW	61.7 ± 1.2 ^a,C^	52.7 ± 0.5 ^b,C^	49.2 ± 0.6 ^c,C^	50.4 ± 0.4 ^d,C^	84.3 ± 1.2 ^a,D^	73.1 ± 0.6 ^b,B^	67.7 ± 0.5 ^c,D^	68.8 ± 0.3 ^d,D^
ACL	25.4 ± 0.8 ^a,C^	21.9 ± 0.3 ^b,C^	17.6 ± 0.3 ^c,C^	18.7 ± 0.4 ^d,C^	29.9 ± 0.4 ^a,D^	24.4 ± 0.3 ^b,A^	20.1 ± 0.4 ^c,A^	20.5 ± 0.5 ^d,A^
IAC	87.1 ± 1.4 ^a,C^	74.6 ± 0.6 ^b,C^	66.8 ± 0.7 ^c,A^	69.1 ± 0.6 ^d,C^	114 ± 1 ^a,D^	97.5 ± 0.7 ^b,D^	87.8 ± 0.6 ^c,C^	89.3 ± 0.6 ^c,D^

The results are presented as means in dry matter ± SD, n = 4 (the mean of four measurements). For each variable and effect, values followed with different letters are significantly different at *p* < 0.05. TE (Trolox equivalent), ACW (antioxidant capacity of water-soluble compounds), ACL (antioxidant capacity of lipid-soluble compounds), IAC (integral antioxidant capacity), S (start of storage experiment), D (storage condition without the presence of sunlight at 23 °C), L (storage condition in the presence of sunlight at 23 °C), T (storage in thermostatic device at 40 °C).

## Data Availability

Data are contained within the article.

## Supplementary Materials

The following supporting information can be downloaded at: https://www.mdpi.com/article/10.3390/antiox12040962/s1, Table S1: Basic chemical analyses; Table S2: Individual phenolic content in free phenolic fraction of the nutraceutical mixtures M1 and M2 under defined storage conditions; Table S3: Individual phenolic content in free phenolic fraction of the nutraceutical mixtures M3 and M4 under defined storage conditions; Table S4: Individual phenolic content in soluble bound phenolic fraction of the nutraceutical mixtures M1 and M2 under defined storage conditions; Table S5: Individual phenolic content in soluble bound phenolic fraction of the nutraceutical mixtures M3 and M4 under defined storage conditions; Table S6: Individual phenolic content in insoluble bound phenolic fraction of the nutraceutical mixtures M1 and M2 under defined storage conditions; Table S7: Individual phenolic content in insoluble bound phenolic fraction of the nutraceutical mixtures M3 and M4 under defined storage conditions; Table S8: Total individual phenolic content of the nutraceutical mixtures M1 and M2 under defined storage conditions; Table S9: Total individual phenolic content of the nutraceutical mixtures M3 and M4 under defined storage conditions; Table S10: Relations of several evaluation parameters of the nutraceutical mixtures with edible flowers evaluated at the start of the storage experiment; Table S11: Relations of several evaluation parameters of the nutraceutical mixtures with edible flowers evaluated under the storage conditions at 23 °C and without the presence of sunlight; Table S12: Relations of several evaluation parameters of the nutraceutical mixtures with edible flowers evaluated under the storage conditions at 23 °C in the presence of sunlight; Table S13: Relations of several evaluation parameters of the nutraceutical mixtures with edible flowers evaluated under the storage conditions at 40 °C in a thermostatic device.
