# Supplementary material for: Influence of Storage Conditions on Stability of Phenolic Compounds and Antioxidant Activity Values in Nutraceutical Mixtures with Edible Flowers as New Dietary Supplements
Source: Antioxidants (Basel). 2023 Apr 19;12(4):962. doi: 10.3390/antiox12040962 (PMC10135932; doi:10.3390/antiox12040962)
Supplement: Supplementary file 1 [file antioxidants-12-00962-s001.zip › antioxidants-2329357-supplementary.pdf]

Supplementary material

Influence of Storage Conditions on Stability of Phenolic Compounds and Antioxidant Activity Values in Nutraceutical Mixtures with Edible Flowers as New Dietary Supplements

Martina Mrázková [1], Daniela Sumczynski [1,*] and Jana Orsavová [2]

[1] Tomas Bata University in Zlín, Department of Food Analysis and Chemistry, Vavrečkova 5669, 760 01 Zlín, Czech Republic
[2] Tomas Bata University in Zlín, Language Centre, Štefánikova 5670, 760 01 Zlín, Czech Republic
* Correspondence: sumczynski@utb.cz

Table S1. Basic chemical analyses.

	S	D	L	T	S
Samples	Dry matter (%)				Ash (%)
M1	91.5±0.14^{a,A}	91.5±0.10^{a,A}	91.6±0.11^{a,A}	94.1±0.18^{a,B}	2.29±0.01^{a}
M2	91.5±0.15^{a,A,C}	91.8±0.13^{b,A}	91.2±0.12^{b,C}	94.1 ±0.14^{a,D}	2.30±0.03^{a}
M3	90.5±0.24^{b,A}	91.1±0.10^{c,B}	90.2±0.18^{c,A}	92.9±0.16^{c,C}	2.55±0.03^{b}
M4	91.1±0.11^{c,A,C}	90.9±0.19^{c,A}	91.3±0.10^{b,C}	93.5±0.10^{d,D}	2.16±0.01^{c}

The results are presented as means in dry matter ± SD (n=5) (the mean value of five measurements). For each variable and effect, values followed with different letters are significantly different at p<0.05. S (start of storage experiment), D (storage condition without the presence of sunlight at 23 °C), L (storage condition in the presence of sunlight at 23 °C), T (storage in thermostatic device at 40 °C).

Table S2. Individual phenolic content in free phenolic fraction of the nutraceutical mixtures M1 and M2 under defined storage conditions.

**Free phenolics (mg/kg)**	**M1S**	**M1D**	**M1L**	**M1T**	**M2S**	**M2D**	**M2L**	**M2T**
E	125±2^{a,A}	102±1^{b,A} (-18%)	95.2±1.2^{c,A} (-24%)	97.1±0.9^{d,A} (-22%)	85.5±1.8^{a,B}	71.2±2.1^{b,B} (-17%)	61.7±0.7^{c,B} (-28%)	64.5±1.4^{d,B} (-25%)
C	9.86±0.48^{a,A}	7.23±0.22^{b,A} (-27%)	6.57±0.21^{c,A} (-33%)	6.77±0.55^{d,A} (-31%)	43.3±0.5^{a,B}	36.2±0.2^{b,B} (-16%)	28.7±0.4^{c,B} (-34%)	32.4±1.1^{d,B} (-25%)
R	1.25±0.06^{a,A}	1.03±0.02^{b,A} (-18%)	0.88±0.05^{c,A} (-30%)	0.92±0.01^{d,A} (-26%)	85.8±0.1^{a,B}	72.3±0.3^{b,B} (-16%)	64.2±0.6^{c,B} (-25%)	67.2±1.2^{d,B} (-57%)
Q	2.46±0.15^{a,A}	1.78±0.31^{b,A} (-28%)	1.51±0.10^{c,A} (-39 %)	1.62±0.12^{d,A} (-34%)	3.70±0.16^{a,B}	2.99±0.22^{b,B} (-19%)	2.62±0.16^{c,B} (-29%)	2.74±0.14^{d,B} (-26%)
NA	64.8±0.5^{a,A}	52.7±0.2^{b,A} (-19%)	45.2±0.4^{c,A} (-30%)	47.8±0.4^{d,A} (-26%)	54.3±0.6^{a,B}	41.7±0.2^{b,B} (-23%)	35.1±0.9^{c,B} (-35%)	37.2±0.8^{d,B} (-31%)
GA	8.05±0.46^{a,A}	6.44±0.31^{b,A} (-20%)	6.03±0.06^{c,A} (-25%)	6.21±0.54^{d,A} (-23%)	14.9±0.5^{a,B}	12.1±0.42^{b,B} (-19%)	9.65±0.4^{c,B} (-35%)	10.9±0.4^{d,B} (-27%)
PA	115±2^{a,A}	89.1±1.3^{b,A} (-23%)	84.1±1.4^{c,A} (-27%)	86.2±1.1^{d,A} (-25%)	128±2^{a,B}	103±1^{b,B} (-20%)	96.7±1.2^{c,B} (-24%)	98.2±1.2^{d,B} (-23%)
p-OH-BA	2.69±0.14^{a,A}	1.99±0.22^{b,A} (-26%)	1.81±0.10^{c,A} (-33%)	1.97±0.10^{b,A} (-27%)	6.14±0.04^{a,B}	5.07±0.01^{b,B} (-17%)	4.72±0.05^{c,B} (-23%)	4.87±0.04^{d,B} (-21%)
CA	14.9±0.5^{a,A}	11.7±0.2^{b,A} (-21%)	10.2±0.3^{c,A} (-25%)	10.9±0.5^{d,A} (-27%)	26.2±0.3^{a,B}	21.7±1.2^{b,B} (-17%)	19.3±0.2^{c,B} (-26%)	20.1±0.3^{d,B} (-23%)
p-CoA	17.0±0.2^{a,A}	14.2±0.4^{b,A} (-16%)	12.8±0.2^{c,A} (-25%)	13.4±0.4^{d,A} (-21%)	3.60±0.10^{a,B}	2.78±0.05^{b,B} (-23%)	2.51±0.07^{c,B} (-20%)	2.63±0.09^{d,B} (-27%)
SA	20.1±0.4^{a,A}	16.7±0.2^{b,A} (-17%)	15.1±0.3^{c,A} (-25%)	15.7±0.4^{d,A} (-22%)	31.6±0.6^{a,B}	24.3±0.4^{b,B} (-22%)	22.5±0.5^{c,B} (-29%)	23.1±0.7^{d,B} (-27%)
EA	2.65±0.13^{a,A}	2.19±0.09^{b,A} (-4%)	1.93±0.10^{c,A} (-27%)	2.02±0.13^{d,A} (-24%)	2.41±0.11^{a,B}	1.86±0.21^{b,B} (-23%)	1.66±0.15^{c,B} (-31%)	1.74±0.10^{d,B} (-28%)

The results are presented as means in dry matter ± SD, n=5 (the mean of five measurements). For each variable and effect, values followed with different letters are significantly different at $p<0.05$. The percentage decreases in the values of free phenolics for individual storage conditions are presented in parentheses. E (epicatechin), C (catechin), R (rutin), Q (quercetin), NA (neochlorogenic acid), GA (gallic acid), PA (protocatechuic acid), *p*-OH-BA (-*p*-hydroxybenzoic acid), CA (caffeic acid), *p*-CoA (*p*-coumaric acid), SA (sinapic acid), EA (ellagic acid), S (start of storage experiment), D (storage condition without the presence of sunlight at 23 °C), L (storage condition in the presence of sunlight at 23 °C), T (storage in the thermostatic device at 40 °C).

Table S3. Individual phenolic content in free phenolic fraction of the nutraceutical mixtures M3 and M4 under defined storage conditions.

Free phenolics (mg/kg)	M3S	M3D	M3L	M3T	M4S	M4D	M4L	M4T
E	117±2^{a,A}	99.1±1.3^{b,A} (-15%)	90.1±1.1^{c,A} (-23%)	92.4±1.4^{d,A} (-21%)	72.6±0.2^{a,B}	61.7±1.1^{b,B} (-15%)	56.2±0.9^{c,B} (-23%)	57.6±1.9^{d,B} (-21%)
C	65.4±1.9^{a,A}	51.7±1.1^{b,A} (-21%)	45.2±1.2^{c,A} (-31%)	47.2±1.2^{d,A} (-28%)	46.4±1.4^{a,B}	34.2±0.8^{b,B} (-26%)	27.4±0.7^{c,B} (-41%)	29.8±0.9^{d,B} (-36%)
R	108±2^{a,A}	87.8±1.2^{b,A} (-19%)	77.4±0.8^{c,A} (-28%)	81.2±1.3^{d,A} (-25%)	82.7±0.6^{a,B}	64.8±0.2^{b,B} (-22%)	57.8±0.4^{c,B} (-30%)	60.2±1.2^{d,B} (-27%)
Q	0.86±0.04^{a,A}	0.71±0.02^{b,A} (-10%)	0.54±0.05^{c,A} (-37%)	0.63±0.02^{d,A} (-27%)	2.60±0.05^{a,B}	2.03±0.02^{b,B} (-22%)	1.76±0.08^{c,B} (-32%)	1.82±0.04^{d,B} (-30%)
NA	51.5±0.4^{a,A}	37.8±0.3^{b,A} (-27%)	33.7±0.7^{c,A} (-35%)	35.4±0.6^{d,A} (-31%)	48.7±0.2^{a,B}	37.1±0.6^{b,A} (-24%)	31.4±0.5^{c,B} (-36%)	34.7±0.2^{d,B} (-29%)
GA	2.32±0.01^{a,A}	1.87±0.02^{b,A} (-19%)	1.52±0.02^{c,A} (-34%)	1.67±0.04^{d,A} (-28%)	8.40±0.16^{a,B}	6.25±0.22^{b,B} (-26%)	5.87±0.10^{c,B} (-30%)	6.01±0.22^{b,B} (-28%)
PA	117±1^{a,A}	87.2±1.3^{b,A} (-25%)	83.4±0.9^{c,A} (-29%)	85.9±1.2^{d,A} (-27%)	103±1^{a,B}	79.8±2.1^{b,B} (-23%)	75.8±1.0^{c,B} (-26%)	77.1±0.9^{d,B} (-25%)
p-OH-BA	7.01±0.12^{a,A}	5.74±0.22^{b,A} (-18%)	5.54±0.10^{c,A} (-21%)	5.64±0.12^{d,A} (-20%)	5.99±0.27^{a,B}	4.47±0.11^{b,B} (-25%)	4.30±0.07^{c,B} (-28%)	4.42±0.22^{d,B} (-26%)
CA	27.5±0.34^{a,A}	21.9±0.27^{b,A} (-21%)	19.5±0.04^{c,A} (-29%)	20.4±0.22^{d,A} (-26%)	24.7±1.09^{a,B}	18.9±0.32^{b,B} (-23%)	16.3±0.08^{c,B} (-34%)	17.2±0.09^{d,B} (-30%)
p-CoA	3.35±0.11^{a,A}	2.46±0.21^{b,A} (-27%)	2.27±0.10^{c,A} (-32%)	2.34±0.10^{d,A} (-30%)	0.98±0.03^{a,B}	0.74±0.02^{b,B} (-24%)	0.61±0.02^{c,B} (-38%)	0.69±0.04^{d,B} (-30%)
SA	25.6±0.10^{a,A}	21.1±0.30^{b,A} (-18%)	19.2±0.07^{c,A} (-25%)	20.1±0.10^{d,A} (-21%)	33.0±0.06^{a,B}	26.5±0.01^{b,B} (-20%)	23.1±0.05^{c,B} (-30%)	24.7±0.08^{d,B} (-25%)
EA	0.88±0.03^{a,A}	0.67±0.08^{b,A} (-24%)	0.57±0.01^{c,A} (-35%)	0.61±0.02^{d,A} (-31%)	0.55±0.02^{a,B}	0.38±0.02^{b,B} (-31%)	0.31±0.02^{c,B} (-44%)	0.34±0.04^{d,B} (-37%)

The results are presented as means in dry matter ± SD, n=5 (the mean of five measurements). For each variable and effect, values followed with different letters are significantly different at $p<0.05$. The percentage decreases in the values of free phenolics for individual storage conditions are presented in parentheses. E (epicatechin), C (catechin), R (rutin), Q (quercetin), NA (neochlorogenic acid), GA (gallic acid), PA (protocatechuic acid), *p*-OH-BA (-*p*-hydroxybenzoic acid), CA (caffeic acid), *p*-CoA (*p*-coumaric acid), SA (sinapic acid), EA (ellagic acid), S (start of storage experiment), D (storage condition without the presence of sunlight at 23 °C), L (storage condition in the presence of sunlight at 23 °C), T (storage in the thermostatic device at 40 °C).

Table S4. Individual phenolic content in soluble bound phenolic fraction of the nutraceutical mixtures M1 and M2 under defined storage conditions.

Soluble bound phenolics (mg/kg)	M1S	M1D	M1L	M1T	M2S	M2D	M2L	M2T
E	182±2^{a,A}	143±4^{b,A}<br>(-21%)	136±3^{c,A}<br>(-25%)	139±1^{d,A}<br>(-24%)	129±2^{a,B}	107±2.1^{b,B}<br>(-17%)	98.6±0.7^{c,B}<br>(-24%)	101±2^{d,B}<br>(-22%)
C	80.7±0.4^{a,A}	64.3±0.2^{b,A}<br>(-20%)	58.3±0.5^{c,A}<br>(-28%)	61.1±0.7^{d,A}<br>(-24%)	48.9±0.1^{a,B}	41.9±0.3^{b,B}<br>(-14%)	36.4±0.2^{c,B}<br>(-26%)	37.9±0.4^{d,B}<br>(-22%)
R	16.2±0.3^{a,A}	12.7±0.1^{b,A}<br>(-22%)	10.8±0.4^{c,A}<br>(-33%)	11.2±0.5^{d,A}<br>(-31%)	17.8±0.3^{a,B}	14.6±0.2^{b,B}<br>(-18%)	11.1±0.2^{c,A}<br>(-38%)	12.0±0.2^{d,B}<br>(-33%)
Q	1.22±0.05^{a,A}	0.88±0.02^{b,A}<br>(-28%)	0.71±0.01^{c,A}<br>(-42%)	0.76±0.02^{d,A}<br>(-38%)	0.19±0.02^{a,B}	0.14±0.01^{b,B}<br>(-26%)	0.10±0.01^{c,B}<br>(-47%)	0.12±0.01^{d,B}<br>(-37%)
NA	11.8±0.3^{a,A}	9.23±0.1^{b,A}<br>(-22%)	8.74±0.12^{c,A}<br>(-26%)	9.01±0.4^{d,A}<br>(-24%)	4.78±0.09^{a,B}	3.97±0.03^{b,B}<br>(-17%)	3.61±0.06^{c,B}<br>(-24%)	3.72±0.05^{d,B}<br>(-22%)
GA	4.18±0.18^{a,A}	3.07±0.11^{b,A}<br>(-27%)	2.71±0.10^{c,A}<br>(-35%)	2.87±0.10^{d,A}<br>(-31%)	58.8±0.2^{a,B}	50.7±0.2^{b,B}<br>(-14%)	45.7±0.2^{c,B}<br>(-22%)	47.2±0.4^{d,B}<br>(-20%)
PA	101±3^{a,A}	83.7±2.3^{b,A}<br>(-17%)	79.8±1.1^{c,A}<br>(-21%)	81.2±1.4^{d,A}<br>(-20%)	101±2^{a,A}	69.9±1.2^{b,B}<br>(-31%)	65.1±0,9^{c,B}<br>(-36%)	67.8±1.0^{d,B}<br>(-33%)
p-OH-BA	0.91±0.05^{a,A}	0.67±0.02^{b,A}<br>(-26%)	0.56±0.04^{c,A}<br>(-38%)	0.59±0.02^{d,A}<br>(-35%)	0.41±0.02^{a,B}	0.28±0.01^{b,B}<br>(-32%)	0.22±0.01^{c,B}<br>(-46%)	0.24±0.02^{d,B}<br>(-41%)
CA	1.62±0.06^{a,A}	1.32±0.02^{b,A}<br>(-19%)	1.23±0.05^{c,A}<br>(-24%)	1.27±0.01^{d,A}<br>(-22%)	3.05±0.11^{a,B}	2.47±0.08^{b,B}<br>(-19%)	2.28±0.12^{c,B}<br>(-25%)	2.32±0.13^{d,B}<br>(-24%)
p-CoA	0.56±0.10^{a,A}	0.47±0.08^{b,A}<br>(-16%)	0.37±0.05^{c,A}<br>(-34%)	0.42±0.01^{d,A}<br>(-25%)	0.27±0.04^{a,B}	0.21±0.01^{b,B}<br>(-22%)	0.16±0.02^{c,B}<br>(-41%)	0.18±0.04^{d,B}<br>(-33%)
SA	3.02±0.02^{a,A}	2.67±0.02^{b,A}	2.52±0.02^{c,A}	2.61±0.02^{d,A}	1.10±0.09^{a,B}	0.92±0.05^{b,B}	0.81±0.01^{c,B}	0.87±0.04^{d,B}

		(-12%)	(-17%)	(-14%)		(-16%)	(-26%)	(-21%)
EA	0.09±0.01^{a,A}	0.07±0.01^{b,A}	0.06±0.01^{b,A}	0.06±0.01^{b,A}	0.13±0.01^{a,B}	0.10±0.02^{b,B}	0.07±0.01^{c,A}	0.08±0.01^{c,B}
		(-22%)	(-3%)	(-3%)		(-23%)	(-46%)	(-38%)

The results are presented as means in dry matter ± SD, n=5 (the mean of five measurements). For each variable and effect, values followed with different letters are significantly different at $p<0.05$. The percentage decreases in the values of soluble bound phenolics for individual storage conditions are presented in parentheses. E (epicatechin), C (catechin), R (rutin), Q (quercetin), NA (neochlorogenic acid), GA (gallic acid), PA (protocatechuic acid), *p*-OH-BA (-*p*-hydroxybenzoic acid), CA (caffeic acid), *p*-CoA (*p*-coumaric acid), SA (sinapic acid), EA (ellagic acid), S (start of storage experiment), D (storage condition without the presence of sunlight at 23 °C), L (storage condition in the presence of sunlight at 23 °C), T (storage in the thermostatic device at 40 °C).

Table S5. Individual phenolic content in soluble bound phenolic fraction of the nutraceutical mixtures M3 and M4 under defined storage conditions.

**Soluble bound phenolics (mg/kg)**	**M3S**	**M3D**	**M3L**	**M3T**	**M4S**	**M4D**	**M4L**	**M4T**
E	117±2^{a,A}	99.7±1.3^{b,A} (-15%)	95.4±1.1^{c,A} (-18%)	97.4±0.8^{d,A} (-17%)	65.6±0.1^{a,B}	53.7±0.3^{b,B} (-18%)	48.2±0.4^{c,B} (-27%)	50.7±07^{d,B} (-23%)
C	118±1^{a,A}	96.3±4^{b,A} (-18%)	92.7±0.9^{c,A} (-21%)	94.0±1.1^{d,A} (-20%)	73.0±0.4^{a,B}	57.2±0.1^{b,B} (-22%)	52.1±0.2^{c,B} (-29%)	54.3±0.6^{d,B} (-26%)
R	33.1±0.1^{a,A}	27.4±0.2^{b,A} (-17%)	23.2±0.1^{c,A} (-30%)	25.3±0.2^{d,A} (-24%)	24.4±0.2^{a,B}	18.9±0.2^{b,B} (-23%)	16.0±0.1^{c,B} (-34%)	17.1±0.4^{d,B} (-30%)
Q	0.87±0.04^{a,A}	0.62±0.01^{b,A} (-29%)	0.52±0.03^{c,A} (-40%)	0.57±0.05^{d,A} (-34%)	0.19±0.03^{a,B}	0.13±0.02^{b,B} (-32%)	0.11±0.01^{c,B} (-42%)	0.13±0.01^{b,B} (-32%)
NA	7.08±0.12^{a,A}	6.02±0.02^{b,A} (-15%)	5.60±0.09^{c,A} (-21%)	5.74±0.11^{d,A} (-19%)	6.60±0.18^{a,B}	5.47±0.12^{b,B} (-17%)	5.03±0.11^{c,B} (-24%)	5.21±0.10^{d,B} (-21%)
GA	2.60±0.09^{a,A}	2.07±0.02^{b,A} (-20%)	1.82±0.05^{c,A} (-30%)	1.99±0.05^{d,A} (-23%)	55.1±0.2^{a,B}	47.4±0.3^{b,B} (-14%)	43.7±0.2^{c,B} (-21%)	45.1±0.6^{d,B} (-18%)
PA	72.2±0.4^{a,A}	57.9±0.2^{b,A} (-20%)	53.2±0.2^{c,A} (-26 %)	55.2±0.4^{d,A} (-24%)	60.1±0.9^{a,B}	47.2±0.7^{b,B} (-21%)	42.1±0.7^{c,B} (-30%)	45.8±0.2^{d,B} (-24%)
p-OH-BA	0.66±0.06^{a,A}	0.47±0.04^{b,A} (-29%)	0.41±0.02^{c,A} (-38%)	0.45±0.04^{d,A} (-32%)	0.58±0.04^{a,B}	0.41±0.01^{b,B} (-29%)	0.33±0.02^{c,B} (-43%)	0.37±0.01^{d,B} (-36%)
CA	2.64±0.05^{a,A}	2.17±0.01^{b,A} (-18%)	2.02±0.01^{c,A} (-23%)	2.10 ±0.04^{d,A} (-20%)	1.05±0.10^{a,B}	0.74±0.02^{b,B} (-30%)	0.67±0.04^{c,B} (-36%)	0.71±0.10^{d,B} (-32%)
p-CoA	0.59±0.03^{a,A}	0.42±0.01^{b,A} (-29%)	0.34±0.01^{c,A} (-42%)	0.38±0.03^{d,A} (-36%)	0.37±0.04^{a,B}	0.29±0.05^{b,B} (-22%)	0.22±0.05^{c,B} (-41%)	0.26±0.04^{d,B} (-30%)
SA	7.34±0.14^{a,A}	6.37±0.10^{b,A} (-13%)	6.03±0.10^{c,A} (-18%)	6.21±0.11^{d,A} (-15%)	6.03±0.01^{a,B}	5.12±0.01^{b,B} (-15%)	4.93±0.02^{c,B} (-18%)	5.01±0.02^{d,B} (-17%)

EA	0.36±0.03[a,A]	0.27±0.04[b,A] (-25%)	0.21±0.02[c,A] (-42%)	0.24±0.02[d,A] (-32%)	0.59±0.04[a]	0.46±0.01[b,B] (-22%)	0.39±0.01[c,B] (-34%)	0.42±0.01[d,B] (-29%)

The results are presented as means in dry matter ± SD, n=5 (the mean of five measurements). For each variable and effect, values followed with different letters are significantly different at $p<0.05$. The percentage decreases in the values of soluble bound phenolics for individual storage conditions are presented in parentheses. E (epicatechin), C (catechin), R (rutin), Q (quercetin), NA (neochlorogenic acid), GA (gallic acid), PA (protocatechuic acid), *p*-OH-BA (-*p*-hydroxybenzoic acid), CA (caffeic acid), *p*-CoA (*p*-coumaric acid), SA (sinapic acid), EA (ellagic acid), S (start of storage experiment), D (storage condition without the presence of sunlight at 23 °C), L (storage condition in the presence of sunlight at 23 °C), T (storage in the thermostatic device at 40 °C).

Table S6. Individual phenolic content in insoluble bound phenolic fraction of the nutraceutical mixtures M1 and M2 under defined storage conditions.

Insoluble bound phenolics (mg/kg)	M1S	M1D	M1L	M1T	M2S	M2D	M2L	M2T
E	93.1±1.3^{a,A}	75.3±1.1^{b,A} (-19%)	71.1±0.6^{c,A} (-24%)	72.1±0.8^{d,A} (-23%)	116±2^{a,B}	97.2±1.7^{b,B} (-16%)	93.9±0.9^{c,B} (-19%)	95.1±1.0^{d,B} (-18%)
C	6.54±0.09^{a,A}	5.05±0.06^{b,A} (-23%)	4.75±0.04^{c,A} (-27%)	4.89±0.05^{d,A} (-25%)	6.89±0.16^{a,B}	5.78±0.21^{b,B} (-16%)	5.50±0.10^{c,B} (-20%)	5.61±0.16^{d,B} (-19%)
R	0.70±0.05^{a,A}	0.49±0.04^{b,A} (-30%)	0.41±0.01^{c,A} (-41%)	0.44±0.02^{d,A} (-37%)	5.33±0.06^{a,B}	4.47±0.04^{b,B} (-16%)	4.03±0.02^{c,B} (-24%)	4.20±0.05^{d,B} (-21%)
Q	1.09±0.05^{a,A}	0.77±0.02^{b,A} (-29%)	0.68±0.02^{c,A} (-38%)	0.71±0.01^{d,A} (-35%)	1.30±0.03^{a,B}	1.01±0.02^{b,B} (-22%)	0.89±0.01^{c,B} (-32%)	0.92±0.04^{d,B} (-29%)
NA	26.4±0.2^{a,A}	21.8±0.2^{b,A} (-17%)	19.3±0.2^{c,A} (-27%)	20.2±0.5^{d,A} (-23%)	28.2±0.1^{a,B}	22.3±0.2^{b,B} (-21%)	20.1±0.3^{c,B} (-29%)	21.3±0.2^{d,B} (-24%)
GA	0.47±0.04^{a,A}	0.38±0.02^{b,A} (-19%)	0.31±0.02^{c,A} (-34%)	0.33±0.06^{c,A} (-30%)	0.56±0.01^{a,B}	0.41±0.01^{b,B} (-27%)	0.34±0.01^{c,A} (-39%)	0.37±0.04^{d,A} (-34%)
PA	4.01±0.06^{a,A}	3.27±0.01^{b,A} (-18%)	3.01±0.04^{c,A} (-25%)	3.07±0.04^{d,A} (-23%)	3.63±0.08^{a,B}	3.02±0.06^{b,B} (-17%)	2.88±0.04^{c,B} (-39%)	2.98±0.04^{d,B} (-18%)
p-OH-BA	1.61±0.08^{a,A}	1.21±0.07^{b,A} (-25%)	1.01±0.02^{c,A} (-37%)	1.07±0.09^{d,A} (-34%)	1.42±0.06^{a,B}	1.07±0.04^{b,B} (-25%)	0.95±0.02^{c,B} (-33%)	0.95±0.07^{c,B} (-33%)
CA	4.86±0.10^{a,A}	3.74±0.08^{b,A} (-23%)	3.57±0.08^{c,A} (-20%)	3.62±0.09^{d,A} (-26%)	5.18±0.09^{a,B}	4.03±0.02^{b,B} (-22%)	3.82±0.04^{c,B} (-26%)	3.99±0.10^{b,B} (-23%)
p-CoA	0.29±0.04^{a,A}	0.21±0.04^{b,A} (-28%)	0.17±0.02^{c,A} (-41%)	0.19±0.02^{d,A} (-34%)	0.36±0.03^{a,B}	0.29±0.04^{b,B} (-28%)	0.27±0.01^{c,B} (-25%)	0.27±0.04^{c,B} (-25%)
SA	5.72±0.19^{a,A}	4.87±0.11^{b,A} (-15%)	4.78±0.10^{c,A} (-16%)	4.81±0.08^{d,A} (-16%)	7.59±0.07^{a,B}	6.47±0.05^{b,B} (-15%)	6.34±0.02^{c,B} (-16%)	6.39±0.05^{c,B} (-16%)

EA	2.19±0.06[a,A]	1.74±0.02[b,A] (-21%)	1.67±0.04[c,A] (-24%)	1.71±0.02[d,A] (-22%)	2.52±0.07[a,B]	1.99±0.04[b,B] (-21%)	1.81±0.05[c,B] (-28%)	1.87±0.08[c,B] (-26%)

The results are presented as means in dry matter ± SD, n=5 (the mean of five measurements). For each variable and effect, values followed with different letters are significantly different at $p<0.05$. The percentage decreases in the values of insoluble bound phenolics for individual storage conditions are presented in parentheses. E (epicatechin), C (catechin), R (rutin), Q (quercetin), NA (neochlorogenic acid), GA (gallic acid), PA (protocatechuic acid), *p*-OH-BA (-*p*-hydroxybenzoic acid), CA (caffeic acid), *p*-CoA (*p*-coumaric acid), SA (sinapic acid), EA (ellagic acid), S (start of storage experiment), D (storage condition without the presence of sunlight at 23 °C), L (storage condition in the presence of sunlight at 23 °C), T (storage in the thermostatic device at 40 °C).

Table S7. Individual phenolic content in insoluble bound phenolic fraction of the nutraceutical mixtures M3 and M4 under defined storage conditions.

**Insoluble bound phenolics (mg/kg)**	**M3S**	**M3D**	**M3L**	**M3T**	**M4S**	**M4D**	**M4L**	**M4T**
E	20.4±0.5^{a,A}	16.3±0.2^{b,A} (-20%)	12.7±0.4^{c,A} (-38%)	14.2±0.4^{d,A} (-30%)	30.7±0.3^{a,B}	24.2±0.1^{b,B} (-21%)	21.3±0.4^{c,B} (-31%)	22.7±0.5^{d,B} (-26%)
C	11.3±0.1^{a,A}	9.22±0.1^{b,A} (-18%)	8.87±0.1^{c,A} (-22%)	9.02±0.2^{d,A} (-20%)	10.4±0.3^{a,B}	8.87±0.2^{b,B} (-15%)	7.82±0.2^{c,B} (-25%)	8.12±0.2^{d,A} (-22%)
R	5.82±0.09^{a,A}	4.27±0.04^{b,A} (-27%)	3.99±0.02^{c,A} (-31%)	4.07±0.04^{d,A} (-30%)	2.71±0.05^{a,B}	2.08±0.01^{b,B} (-23%)	1.81±0.04^{c,B} (-33%)	1.87±0.04^{c,B} (-31%)
Q	0.49±0.06^{a,A}	0.37±0.01^{b,A} (-24%)	0.28±0.02^{c,A} (-43%)	0.31±0.05^{d,A} (-37%)	1.35±0.06^{a,B}	1.06±0.02^{b,B} (-21%)	0.87±0.05^{c,B} (-36%)	0.99±0.07^{d,B} (-27%)
NA	26.0±0.3^{a,A}	20.7±0.3^{b,A} (-20%)	18.3±0.2^{c,A} (-30%)	19.9±0.4^{d,A} (-23%)	63.1±0.3^{a,B}	52.8±0.1^{b,B} (-16%)	48.9±0.2^{c,B} (-23%)	50.3±0.5^{d,B} (-20%)
GA	0.50±0.04^{a,A}	0.38±0.01^{b,A} (-24%)	0.31±0.02^{c,A} (-38%)	0.34±0.06^{d,A} (-32%)	3.30±0.02^{a,B}	2.89±0.03^{b,B} (-12%)	2.62±0.01^{c,B} (-21%)	2.74±0.04^{d,B} (-17%)
PA	18.9±0.3^{a,A}	14.7±0.2^{b,A} (-22%)	12.1±0.1^{c,A} (-36%)	12.7±0.07^{d,A} (-3%)	22.3±0.17^{a,B}	17.1±0.12^{b,B} (-23%)	15.2±0.10^{c,B} (-32%)	15.9±0.11^{d,B} (-29%)
p-OH-BA	0.61±0.03^{a,A}	0.48±0.01^{b,A} (-21%)	0.37±0.01^{c,A} (-39%)	0.42±0.04^{d,A} (-31%)	3.12±0.10^{a,B}	2.47±0.01^{b,B} (-21%)	2.01±0.07^{c,B} (-36%)	2.12±0.04^{d,B} (-32%)
CA	3.51±0.06^{a,A}	2.47±0.04^{b,A} (-30 %)	2.34±0.04^{c,A} (-33%)	2.41±0.04^{d,A} (-31%)	2.85±0.02^{a,B}	2.07±0.01^{b,B} (-27%)	1.91±0.04^{c,B} (-33%)	1.97±0.04^{c,B} (-31%)
p-CoA	0.20±0.02^{a,A}	0.14±0.01^{b,A} (-30%)	0.11±0.01^{c,A} (-45%)	0.12±0.01^{c,A} (-40%)	0.10±0.02^{a,B}	0.07±0.01^{b,B} (-30%)	0.06±0.01^{b,B} (-40%)	0.06±0.01^{b,B} (-40%)
SA	5.03±0.06^{a,A}	4.02±0.01^{b,A} (-20%)	3.91±0.01^{c,A} (-22%)	3.97±0.04^{d,A} (-21%)	1.39±0.08^{a,B}	1.07±0.04^{b,B} (-23%)	0.99±0.02^{c,B} (-29%)	1.02±0.09^{c,B} (-27%)

EA	0.63±0.06[a,A]	0.47±0.08[b,A] (-25%)	0.38±0.01[c,A] (40%)	0.42±0.05[d,A] (-33%)	1.27±0.09[a,B]	0.97±0.02[b,B] (-24%)	0.98±0.04[b,B] (-23%)	0.91±0.05[c,B] (-29%)

The results are presented as means in dry matter ± SD, n=5 (the mean of five measurements). For each variable and effect, values followed with different letters are significantly different at $p<0.05$. The percentage decreases in the values of insoluble bound phenolics for individual storage conditions are presented in parentheses. E (epicatechin), C (catechin), R (rutin), Q (quercetin), NA (neochlorogenic acid), GA (gallic acid), PA (protocatechuic acid), *p*-OH-BA (-*p*-hydroxybenzoic acid), CA (caffeic acid), *p*-CoA (*p*-coumaric acid), SA (sinapic acid), EA (ellagic acid), S (start of storage experiment), D (storage condition without the presence of sunlight at 23 °C), L (storage condition in the presence of sunlight at 23 °C), T (storage in the thermostatic device at 40 °C).

Table S8. Total individual phenolic content of the nutraceutical mixtures M1 and M2 under defined storage conditions.

Total phenolics (mg/kg)	M1S	M1D	M1L	M1T	M2S	M2D	M2L	M2T
E	400±3^{a,A}	320±4^{b,A} (-20%)	302±3^{c,A} (-25%)	308±2^{d,A} (-23%)	331±3^{a,B}	275±3^{b,B} (-17%)	254±1^{c,B} (-23%)	261±3^{d,B} (-21%)
C	97.1±0.6^{a,A}	76.6±0.3^{b,A} (-21%)	69.6±0.5^{c,A} (-28%)	72.8±0.9^{d,A} (-25%)	99.1±0.5^{a,B}	83.9±0.4^{b,B} (-15%)	70.6±0.5^{c,B} (-29%)	75.9±1.2^{d,B} (-23%)
R	18.2±0.3^{a,A}	14.2±0.1^{b,A} (-22%)	12.1±0.4^{c,A} (-34%)	12.6±0.5^{d,A} (-31%)	109±1^{a,B}	91.4±0.4^{b,B} (-16%)	79.3±0.4^{c,B} (-27%)	83.4±1.2^{d,B} (-23%)
Q	4.77±0.2^{a,A}	3.43±0.3^{b,A} (-28%)	2.90±0.1^{c,A} (-39%)	3.09±0.12^{d,A} (-35%)	5.19±0.16^{a,B}	4.14±0.22^{b,B} (-20%)	3.61±0.16^{c,B} (-30%)	3.78±0.15^{d,B} (-27%)
NA	103±1^{a,A}	83.7±0.3^{b,A} (-19%)	73.2±0.5^{c,A} (-29%)	77.0±0.8^{d,A} (-25%)	87.3±0.6^{a,B}	68.0±0.2^{b,B} (-22%)	58.9±1.0^{c,B} (-33%)	62.2±0.8^{d,B} (-29%)
GA	12.7±0.5^{a,A}	9.89±0.33^{b,A} (-22%)	9.05±0.12^{c,A} (-29%)	9.41±0.55^{d,A} (-26%)	74.3±0.5^{a,B}	63.2±0.5^{b,B} (-15%)	55.7±0.4^{c,B} (-25%)	58.5±0.6^{d,B} (-21%)
PA	220±4^{a,A}	176±3^{b,A} (-20%)	167±2^{c,A} (-24%)	171±2^{d,A} (-22%)	233±3^{a,B}	176±2^{b,A} (-24%)	165±2^{c,A} (-29%)	169±2^{d,A} (-27%)
p-OH-BA	5.21±0.20^{a,A}	3.87±0.23^{b,A} (-26%)	3.38±0.11^{c,A} (-35%)	3.63±0.14^{d,A} (-30%)	7.97±0.07^{a,B}	6.42±0.04^{b,B} (-19%)	5.89±0.05^{c,B} (-26%)	6.06±0.08^{d,B} (-24%)
CA	21.4±0.5^{a,A}	16.8±0.2^{b,A} (-21%)	15.0±0.3^{c,A} (-30%)	15.8±0.5^{d,A} (-26%)	34.4±0.3^{a,B}	28.2±1.2^{b,B} (-18%)	25.4±0.2^{c,B} (-26%)	26.4±0.3^{d,B} (-23%)
p-CoA	17.9±0.2^{a,A}	14.9±0.4^{b,A} (-17%)	13.3±0.2^{c,A} (-26%)	14.0±0.4^{d,A} (-22%)	4.23±0.11^{a,B}	3.28±0.06^{b,B} (-22%)	2.94±0.07^{c,B} (-30%)	3.08±0.11^{d,B} (-27%)
SA	28.9±0.4^{a,A}	24.2±0.2^{b,A} (-16%)	22.4±0.3^{c,A} (-22%)	23.1±0.4^{d,A} (-20%)	40.3±0.6^{a,B}	31.7±0.4^{b,B} (-21%)	29.7±0.5^{c,B} (-26%)	30.4±0.7^{d,B} (-25%)
EA	4.93±0.14^{a,A}	4.00±0.09^{b,A}	3.66±0.11^{c,A}	3.79±0.13^{d,A}	5.06±0.13^{a,B}	3.95±0.21^{b,A}	3.54±0.16^{c,B}	3.69±0.13^{d,B}

		(-19%)	(-26%)	(-23%)		(-22%)	(-30%)	(-27%)

The results are presented as means in dry matter ± SD, n=5 (the mean of five measurements). For each variable and effect, values followed with different letters are significantly different at $p<0.05$. The percentage decreases in the values of total phenolics for individual storage conditions are presented in parentheses. E (epicatechin), C (catechin), R (rutin), Q (quercetin), NA (neochlorogenic acid), GA (gallic acid), PA (protocatechuic acid), *p*-OH-BA (-*p*-hydroxybenzoic acid), CA (caffeic acid), *p*-CoA (*p*-coumaric acid), SA (sinapic acid), EA (ellagic acid), S (start of storage experiment), D (storage condition without the presence of sunlight at 23 °C), L (storage condition in the presence of sunlight at 23 °C), T (storage in the thermostatic device at 40 °C).

Table S9. Total individual phenolic content of the nutraceutical mixtures M3 and M4 under defined storage conditions.

Total phenolics (mg/kg)	M3S	M3D	M3L	M3T	M4S	M4D	M4L	M4T
E	254±3^{a,A}	215±2^{b,A} (-15%)	198±2^{c,A} (-22%)	204±2^{d,A} (-20%)	169±1^{a,B}	140±1^{b,B} (-17%)	126±1^{c,B} (-25%)	131±2^{d,B} (-22%)
C	195±2^{a,A}	157±4^{b,A} (-19%)	147±2^{c,A} (-25%)	150±2^{d,A} (-23%)	130±1^{a,B}	100±1^{b,B} (-23%)	87.3±0.8^{c,B} (-33%)	92.2±1.1^{d,B} (-29%)
R	147±2^{a,A}	119±1^{b,A} (-19%)	105±1^{c,A} (-29%)	111±1^{d,A} (-24%)	110±1^{a,B}	85.8±0.3^{b,B} (-22%)	75.6±0.4^{c,B} (-31%)	79.2±1.3^{d,B} (-28%)
Q	2.22±0.08^{a,A}	1.70±0.02^{b,A} (-23%)	1.34±0.18^{c,A} (-40%)	1.51±0.07^{d,A} (-32%)	4.14±0.08^{a,B}	3.22±0.03^{b,B} (-22%)	2.74±0.09^{c,B} (-34%)	2.94±0.08^{d,B} (-29%)
NA	84.6±0.5^{a,A}	64.5±0.4^{b,A} (-24%)	57.6±0.7^{c,A} (-32%)	61.0±0.7^{d,A} (-28%)	118±1^{a,B}	95.4±0.6^{b,B} (-19%)	85.3±0.5^{c,B} (-28%)	90.2±0.5^{d,B} (-24%)
GA	5.42±0.10^{a,A}	4.32±0.03^{b,A} (-20%)	3.65±0.06^{c,A} (-33%)	4.00±0.09^{d,A} (-26%)	66.8±0.3^{a,B}	56.5±0.4^{b,B} (-15%)	52.2±0.2^{c,B} (-22%)	53.9±0.6^{d,B} (-19%)
PA	208±1^{a,A}	160±1^{b,A} (-23%)	149±1^{c,A} (-28%)	154±1^{d,A} (-26%)	185±1^{a,B}	144±2^{b,B} (-22%)	133±1^{c,B} (-28%)	139±1^{d,B} (-25%)
p-OH-BA	8.28±0.14^{a,A}	6.69±0.22^{b,A} (-19%)	6.32±0.10^{c,A} (-24%)	6.51±0.13^{d,A} (-21%)	9.69±0.30^{a,B}	7.35±0.11^{b,B} (-24%)	6.64±0.07^{c,B} (-31%)	6.91±0.22^{d,B} (-29%)
CA	33.7±0.3^{a,A}	26.5±0.3^{b,A} (-21%)	23.9±0.1^{c,A} (-29%)	24.9±0.2^{d,A} (-26%)	28.6±1.0^{a,B}	21.7±0.3^{b,B} (-24%)	18.9±0.1^{c,B} (-34%)	20.0±0.1^{d,B} (-30%)
p-CoA	4.14±0.11^{a,A}	3.02±0.21^{b,A} (-27%)	2.72±0.10^{c,A} (-34%)	2.84±0.10^{d,A} (-31%)	1.45±0.05^{a,B}	1.10±0.05^{b,B} (-24%)	0.89±0.05^{c,B} (-39%)	1.01±0.06^{d,B} (-30%)
SA	38.0±0.2^{a,A}	31.5±0.3^{b,A} (-17%)	29.1±0.1^{c,A} (-23%)	30.3±0.2^{d,A} (-20%)	40.4±0.1^{a,B}	32.7±0.1^{b,B} (-19%)	29.0±0.1^{c,A} (-28%)	30.7±0.1^{d,B} (-24%)
EA	1.87±0.07^{a,A}	1.41±0.12^{b,A}	1.16±0.02^{c,A}	1.27±0.06^{d,A}	2.41±0.10^{a,B}	1.81±0.03^{b,B}	1.68±0.05^{c,B}	1.67±0.06^{d,B}

		(-25%)	(-38%)	(-32%)		(-25%)	(-30%)	(-31%)

The results are presented as means in dry matter ± SD, n=5 (the mean of five measurements). For each variable and effect, values followed with different letters are significantly different at $p<0.05$. The percentage decreases in the values of total phenolics for individual storage conditions are presented in parentheses. E (epicatechin), C (catechin), R (rutin), Q (quercetin), NA (neochlorogenic acid), GA (gallic acid), PA (protocatechuic acid), *p*-OH-BA (-*p*-hydroxybenzoic acid), CA (caffeic acid), *p*-CoA (*p*-coumaric acid), SA (sinapic acid), EA (ellagic acid), S (start of storage experiment), D (storage condition without the presence of sunlight at 23 °C), L (storage condition in the presence of sunlight at 23 °C), T (storage in the thermostatic device at 40 °C).

Table S10. Relations of several evaluation parameters of the nutraceutical mixtures with edible flowers evaluated at the start of the storage experiment.

S	f-ABTS	f-DPPH	sb-ABTS	sb-DPPH	ib-ABTS	ib-DPPH	t-ABTS	t-DPPH	ACW	ACL	IAC
f-TPC	0.7256	0.4755	*	*	*	*	*	*	*	*	*
sb-TPC	*	*	-0.8856	0.4350	*	*	*	*	*	*	*
ib-TPC	*	*	*	*	0.8341	0.8203	*	*	*	*	*
t-TPC	*	*	*	*	*	*	0.6207	0.7657	0.8543	0.1307	0.6599
f-E	-0.8680	-0.1917	*	*	*	*	*	*	*	*	*
sb-E	*	*	0.7884	-0.0788	*	*	*	*	*	*	*
ib-E	*	*	*	*	0.2059	0.1715	*	*	*	*	*
t-E	*	*	*	*	*	*	-0.3488	-0.6763	-0.6800	0.1646	-0.4201
f-C	0.4915	0.7863	*	*	*	*	*	*	*	*	*
sb-C	*	*	0.1487	0.9596	*	*	*	*	*	*	*
ib-C	*	*	*	*	-0.1086	-0.0379	*	*	*	*	*
t-C	*	*	*	*	*	*	-0.3482	0.0611	-0.0841	-0.5419	-0.2719
f-R	0.6609	0.6866	*	*	*	*	*	*	*	*	*
sb-R	*	*	-0.1289	0.7425	*	*	*	*	*	*	*
ib-R	*	*	*	*	0.0115	0.1682	*	*	*	*	*
t-R	*	*	*	*	*	*	0.3815	0.7017	0.5547	0.1334	0.4508
f-Q	0.5658	-0.7069	*	*	*	*	*	*	*	*	*
sb-Q	*	*	0.5224	0.5795	*	*	*	*	*	*	*
ib-Q	*	*	*	*	0.8608	0.7816	*	*	*	*	*
t-Q	*	*	*	*	*	*	0.4816	0.0802	0.2128	0.6555	0.4089
f-NA	-0.6869	-0.7788	*	*	*	*	*	*	*	*	*
sb-NA	*	*	0.1776	0.4125	*	*	*	*	*	*	*
ib-NA	*	*	*	*	0.6977	0.6510	*	*	*	*	*
t-NA	*	*	*	*	*	*	0.0793	0.0625	0.2638	-0.2697	0.0785
f-GA	0.6263	0.7100	*	*	*	*	*	*	*	*	*

sb-GA	*	*	-0.5083	-0.7509	*	*	*	*	*	*	*
ib-GA	*	*	*	*	0.6763	0.6307	*	*	*	*	*
t-GA	*	*	*	*	*	*	0.9701	0.8365	0.9057	0.7923	0.9584
f-PA	0.2516	-0.5672	*	*	*	*	*	*	*	*	*
sb-PA	*	*	0.7548	-0.5678	*	*	*	*	*	*	*
ib-PA	*	*	*	*	0.0893	0.1237	*	*	*	*	*
t-PA	*	*	*	*	*	*	0.0547	-0.2379	-0.3189	0.5624	-0.0040
f-*p*-OH-BA	0.6653	0.6817	*	*	*	*	*	*	*	*	*
sb-*p*-OH-BA	*	*	0.1540	0.5797	*	*	*	*	*	*	*
ib-*p*-OH-BA	*	*	*	*	0.7563	0.6650	*	*	*	*	*
t-*p*-OH-BA	*	*	*	*	*	*	0.6119	0.8816	0.8517	0.1697	0.6753
f-CA	0.7261	0.6078	*	*	*	*	*	*	*	*	*
sb-CA	*	*	0.7796	-0.3053	*	*	*	*	*	*	*
ib-CA	*	*	*	*	-0.1150	-0.1266	*	*	*	*	*
t-CA	*	*	*	*	*	*	0.5527	0.7294	0.5625	0.4848	0.5955
f-*p*-CoA	-0.7954	-0.6580	*	*	*	*	*	*	*	*	*
sb-*p*-CoA	*	*	0.3068	0.8731	*	*	*	*	*	*	*
ib-*p*-CoA	*	*	*	*	-0.1357	-0.1172	*	*	*	*	*
t-*p*-CoA	*	*	*	*	*	*	-0.6663	-0.9174	-0.8457	0.5937	0.9478
f-SA	0.9393	0.3135	*	*	*	*	*	*	*	*	*
sb-SA	*	*	-0.4004	0.8894	*	*	*	*	*	*	*
ib-SA	*	*	*	*	-0.2870	-0.2374	*	*	*	*	*
t-SA	*	*	*	*	*	*	0.7832	0.9664	0.8967	0.4821	0.8315
f-EA	-0.1984	-0.9725	*	*	*	*	*	*	*	*	*
sb-EA	*	*	-0.8541	0.4156	*	*	*	*	*	*	*
ib-EA	*	*	*	*	0.3547	0.2969	*	*	*	*	*
t-EA	*	*	*	*	*	*	0.1750	-0.2513	-0.1902	0.5789	0.0922

t-TAC	*	*	*	*	*	*	0.8798	0.7377	0.6709	0.9691	0.8627
D3G	*	*	*	*	*	*	0.9954	0.8626	0.8848	0.8927	0.9839
C3G	*	*	*	*	*	*	-0.7867	-0.8227	-0.7003	-0.7676	-0.8050
C3R	*	*	*	*	*	*	0.9565	0.9636	0.9222	0.7930	0.9722
P3G	*	*	*	*	*	*	0.2192	0.2997	0.0933	0.4240	0.2373
Pe3G	*	*	*	*	*	*	-0.4279	-0.0534	-0.2202	-0.5198	-0.3600
Delphinidin	*	*	*	*	*	*	0.7632	0.8230	0.6942	0.7300	0.7859
Cyanidin	*	*	*	*	*	*	0.3271	0.1196	0.3321	0.1468	0.2917
Pelargo-nidin	*	*	*	*	*	*	0.2833	-0.0986	-0.1070	0.7256	0.2106
Peonidin	*	*	*	*	*	*	0.7174	0.4622	0.4090	0.9710	0.6764
Malvidin	*	*	*	*	*	*	0.6097	0.7804	0.8558	0.1122	0.6538

S (start of the storage experiment), f (free), sb (soluble bound), ib (insoluble bound), TPC (total phenolic content), TAC (total anthocyanin content), E (epicatechin), C (catechin), R (rutin), Q (quercetin), NA (neochlorogenic acid), GA (gallic acid), PA (protocatechuic acid), *p*-OH-BA (*p*-hydroxybenzoic acid), CA (caffeic acid), *p*-CoA (*p*-coumaric acid), SA (sinapic acid), EA (ellagic acid), D3G (delphinidin-3-glucoside), C3G (cyanidin-3-glucoside), C3R (cyanidin-3-rutinoside), P3G (pelargonidin-3-glucoside), Pe3G (peonidin-3-glucoside).

Table S11. Relations of several evaluation parameters of the nutraceutical mixtures with edible flowers evaluated under the storage conditions at 23 °C and without the presence of sunlight.

D	f-ABTS	f-DPPH	sb-ABTS	sb-DPPH	ib-ABTS	ib-DPPH	t-ABTS	t-DPPH	ACW	ACL	IAC
f-TPC	0.9412	0.9790	*	*	*	*	*	*	*	*	*
sb-TPC	*	*	-0.8990	0.4423	*	*	*	*	*	*	*
ib-TPC	*	*	*	*	0.7040	0.7585	*	*	*	*	*
t-TPC	*	*	*	*	*	*	0.4781	0.8738	0.7281	-0.0268	0.5084
f-E	-0.8731	-0.9926	*	*	*	*	*	*	*	*	*
sb-E	*	*	0.8362	-0.0677	*	*	*	*	*	*	*
ib-E	*	*	*	*	0.2373	0.2361	*	*	*	*	*
t-E	*	*	*	*	*	*	-0.1339	-0.8047	-0.5276	0.3530	-0.2365
f-C	0.2058	0.3413	*	*	*	*	*	*	*	*	*
sb-C	*	*	0.1922	0.9438	*	*	*	*	*	*	*
ib-C	*	*	*	*	-0.0218	0.0425	*	*	*	*	*
t-C	*	*	*	*	*	*	-0.4989	-0.0310	-0.2543	-0.5316	-0.3917
f-R	0.3922	0.5037	*	*	*	*	*	*	*	*	*
sb-R	*	*	-0.0550	0.7372	*	*	*	*	*	*	*
ib-R	*	*	*	*	0.0735	0.3003	*	*	*	*	*
t-R	*	*	*	*	*	*	0.2113	0.5048	0.4225	0.0594	0.3264
f-Q	0.8601	0.5888	*	*	*	*	*	*	*	*	*
sb-Q	*	*	0.5137	0.5643	*	*	*	*	*	*	*
ib-Q	*	*	*	*	0.9302	0.8429	*	*	*	*	*
t-Q	*	*	*	*	*	*	0.7643	0.2319	0.5147	0.7836	0.6780
f-NA	-0.3838	-0.6537	*	*	*	*	*	*	*	*	*
sb-NA	*	*	0.1577	0.4563	*	*	*	*	*	*	*
ib-NA	*	*	*	*	0.6808	0.5752	*	*	*	*	*
t-NA	*	*	*	*	*	*	0.0623	0.3980	0.2287	-0.3150	0.0368

f-GA	0.8232	0.4785	*	*	*	*	*	*	*	*	*
sb-GA	*	*	-0.4892	-0.7516	*	*	*	*	*	*	*
ib-GA	*	*	*	*	0.6615	0.5590	*	*	*	*	*
t-GA	*	*	*	*	*	*	0.9668	0.8304	0.9704	0.7003	0.9708
f-PA	0.4842	-0.0137	*	*	*	*	*	*	*	*	*
sb-PA	*	*	0.7262	-0.2834	*	*	*	*	*	*	*
ib-PA	*	*	*	*	0.0582	0.0660	*	*	*	*	*
t-PA	*	*	*	*	*	*	0.0404	-0.6951	-0.3693	0.5332	-0.0517
f-*p*-OH-BA	0.3705	0.4480	*	*	*	*	*	*	*	*	*
sb-*p*-OH-BA	*	*	0.1395	0.5518	*	*	*	*	*	*	*
ib-*p*-OH-BA	*	*	*	*	0.7587	0.6099	*	*	*	*	*
t-*p*-OH-BA	*	*	*	*	*	*	0.4272	0.8426	0.7147	0.0652	0.5368
f-CA	0.4963	0.5205	*	*	*	*	*	*	*	*	*
sb-CA	*	*	0.8184	-0.2481	*	*	*	*	*	*	*
ib-CA	*	*	*	*	-0.0303	-0.0125	*	*	*	*	*
t-CA	*	*	*	*	*	*	0.4655	0.3622	0.4765	0.4999	0.5401
f-*p*-CoA	-0.5602	-0.7589	*	*	*	*	*	*	*	*	*
sb-*p*-CoA	*	*	0.2678	0.7633	*	*	*	*	*	*	*
ib-*p*-CoA	*	*	*	*	-0.0207	-0.0584	*	*	*	*	*
t-*p*-CoA	*	*	*	*	*	*	0.4245	-0.3248	0.0491	0.8290	0.3648
f-SA	0.7826	0.9670	*	*	*	*	*	*	*	*	*
sb-SA	*	*	-0.3938	0.9025	*	*	*	*	*	*	*
ib-SA	*	*	*	*	-0.2469	-0.1414	*	*	*	*	*
t-SA	*	*	*	*	*	*	0.5075	0.8222	0.7475	0.1957	0.6121
f-EA	0.0030	-0.4516	*	*	*	*	*	*	*	*	*
sb-EA	*	*	-0.8717	0.3922	*	*	*	*	*	*	*
ib-EA	*	*	*	*	0.3653	0.3194	*	*	*	*	*

t-EA	*	*	*	*	*	*	0.3435	-0.3800	-0.0472	0.6572	0.2269
t-TAC	*	*	*	*	*	*	0.8904	0.3127	0.6484	0.9958	0.8588
D3G	*	*	*	*	*	*	0.9853	0.6036	0.8605	0.9164	0.9786
C3G	*	*	*	*	*	*	-0.6496	-0.5910	-0.6971	-0.5666	-0.7238
C3R	*	*	*	*	*	*	0.9897	0.6492	0.8885	0.8912	0.9886
P3G	*	*	*	*	*	*	0.4834	0.0340	0.2973	0.7308	0.5037
Pe3G	*	*	*	*	*	*	-0.5670	-0.1408	-0.3520	-0.5477	-0.4678
Delphinidin	*	*	*	*	*	*	0.8167	0.4232	0.6838	0.8750	0.8366
Cyanidin	*	*	*	*	*	*	0.3982	0.1657	0.2726	0.2838	0.3060
Pelargonidin	*	*	*	*	*	*	0.5265	-0.2505	0.1323	0.8595	0.4358
Peonidin	*	*	*	*	*	*	*	*	*	*	*
Malvidin	*	*	*	*	*	*	0.8709	0.3080	0.6205	0.9053	0.8023

D (storage experiment provided at 23 °C without the presence of sunlight), f (free), sb (soluble bound), ib (insoluble bound), TPC (total phenolic content), TAC (total anthocyanin content), E (epicatechin), C (catechin), R (rutin), Q (quercetin), NA (neochlorogenic acid), GA (gallic acid), PA (protocatechuic acid), *p*-OH-BA (*p*-hydroxybenzoic acid), CA (caffeic acid), *p*-CoA (*p*-coumaric acid), SA (sinapic acid), EA (ellagic acid), D3G (delphinidin-3-glucoside), C3G (cyanidin-3-glucoside), C3R (cyanidin-3-rutinoside), P3G (pelargonidin-3-glucoside), Pe3G (peonidin-3-glucoside).

Table S12. Relations of several evaluation parameters of the nutraceutical mixtures with edible flowers evaluated under the storage conditions at 23 °C in the presence of sunlight.

L	f-ABTS	f-DPPH	sb-ABTS	sb-DPPH	ib-ABTS	ib-DPPH	t-ABTS	t-DPPH	ACW	ACL	IAC
f-TPC	0.2392	0.6084	*	*	*	*	*	*	*	*	*
sb-TPC	*	*	-0.9175	0.5459	*	*	*	*	*	*	*
ib-TPC	*	*	*	*	0.5479	0.4714	*	*	*	*	*
t-TPC	*	*	*	*	*	*	-0.0023	0.2272	0.2568	-0.5157	-0.0181
f-E	-0.9792	-0.9030	*	*	*	*	*	*	*	*	*
sb-E	*	*	0.7219	0.3168	*	*	*	*	*	*	*
ib-E	*	*	*	*	0.2789	0.1498	*	*	*	*	*
t-E	*	*	*	*	*	*	-0.2429	-0.4148	-0.4588	0.3613	-0.1850
f-C	-0.0109	0.3481	*	*	*	*	*	*	*	*	*
sb-C	*	*	-0.5691	0.3472	*	*	*	*	*	*	*
ib-C	*	*	*	*	-0.1459	0.0152	*	*	*	*	*
t-C	*	*	*	*	*	*	-0.6756	-0.3145	-0.3636	-0.5941	-0.4868
f-R	0.2746	0.5493	*	*	*	*	*	*	*	*	*
sb-R	*	*	-0.7846	-0.0108	*	*	*	*	*	*	*
ib-R	*	*	*	*	0.0854	0.1986	*	*	*	*	*
t-R	*	*	*	*	*	*	0.0371	0.4596	0.4010	0.0115	0.2864
f-Q	0.7273	0.3164	*	*	*	*	*	*	*	*	*
sb-Q	*	*	0.1187	0.7452	*	*	*	*	*	*	*
ib-Q	*	*	*	*	0.8890	0.8006	*	*	*	*	*
t-Q	*	*	*	*	*	*	0.8159	0.5588	0.5812	0.8077	0.7224
f-NA	-0.5590	-0.8346	*	*	*	*	*	*	*	*	*
sb-NA	*	*	0.1036	0.9334	*	*	*	*	*	*	*
ib-NA	*	*	*	*	0.6477	0.6817	*	*	*	*	*
t-NA	*	*	*	*	*	*	0.3282	0.0297	0.1502	-0.3591	-0.0307
f-GA	0.3999	-0.0717	*	*	*	*	*	*	*	*	*

sb-GA	*	*	0.0509	-0.6437	*	*	*	*	*	*	*
ib-GA	*	*	*	*	0.6219	0.6609	*	*	*	*	*
t-GA	*	*	*	*	*	*	0.9839	0.9543	0.9790	0.6600	0.8430
f-PA	-0.0242	-0.3403	*	*	*	*	*	*	*	*	*
sb-PA	*	*	0.8519	0.2784	*	*	*	*	*	*	*
ib-PA	*	*	*	*	0.0896	0.2196	*	*	*	*	*
t-PA	*	*	*	*	*	*	-0.1289	-0.2450	-0.3052	0.5241	-0.0140
f-*p*-OH-BA	0.1952	0.4817	*	*	*	*	*	*	*	*	*
sb-*p*-OH-BA	*	*	-0.0287	0.9347	*	*	*	*	*	*	*
ib-*p*-OH-BA	*	*	*	*	0.7505	0.7207	*	*	*	*	*
t-*p*-OH-BA	*	*	*	*	*	*	0.3165	0.6464	0.6288	0.0195	0.4509
f-CA	0.2578	0.4530	*	*	*	*	*	*	*	*	*
sb-CA	*	*	0.4845	-0.6735	*	*	*	*	*	*	*
ib-CA	*	*	*	*	-0.0197	-0.1432	*	*	*	*	*
t-CA	*	*	*	*	*	*	0.1816	0.5713	0.4751	0.4802	0.5190
f-*p*-CoA	-0.5696	-0.8104	*	*	*	*	*	*	*	*	*
sb-*p*-CoA	*	*	-0.1480	0.8156	*	*	*	*	*	*	*
ib-*p*-CoA	*	*	*	*	0.1486	0.0654	*	*	*	*	*
t-*p*-CoA	*	*	*	*	*	*	-0.4036	-0.7207	-0.7019	-0.1172	-0.5402
f-SA	0.8436	0.9026	*	*	*	*	*	*	*	*	*
sb-SA	*	*	-0.9389	0.3411	*	*	*	*	*	*	*
ib-SA	*	*	*	*	-0.1995	-0.2679	*	*	*	*	*
t-SA	*	*	*	*	*	*	0.3972	0.7500	0.7059	0.2770	0.6043
f-EA	-0.2639	-0.6962	*	*	*	*	*	*	*	*	*
sb-EA	*	*	-0.9166	0.0827	*	*	*	*	*	*	*
ib-EA	*	*	*	*	0.4086	0.2606	*	*	*	*	*
t-EA	*	*	*	*	*	*	0.3354	0.0472	0.0420	0.6673	0.2872

t-TAC	*	*	*	*	*	*	0.7582	0.7785	0.7324	0.9924	0.8992
D3G	*	*	*	*	*	*	0.8631	0.9135	0.8815	0.9284	0.9800
C3G	*	*	*	*	*	*	-0.4561	-0.7986	-0.7359	-0.4923	-0.7083
C3R	*	*	*	*	*	*	0.9086	0.9589	0.9388	0.8701	0.9981
P3G	*	*	*	*	*	*	0.2266	0.5030	0.3961	0.7402	0.5635
Pe3G	*	*	*	*	*	*	-0.6983	-0.3317	-0.3885	-0.5563	-0.4899
Delphinidin	*	*	*	*	*	*	0.6127	0.8230	0.7505	0.8606	0.8607
Cyanidin	*	*	*	*	*	*	0.5597	0.1409	0.2223	0.3000	0.2740
Pelargonidin	*	*	*	*	*	*	0.4166	0.2992	0.2506	0.8887	0.5192
Peonidin	*	*	*	*	*	*	*	*	*	*	*
Malvidin	*	*	*	*	*	*	0.7996	0.6156	0.6129	0.9129	0.7850

L (storage experiment provided at 23 °C in the presence of sunlight), f (free), sb (soluble bound), ib (insoluble bound), TPC (total phenolic content), TAC (total anthocyanin content), E (epicatechin), C (catechin), R (rutin), Q (quercetin), NA (neochlorogenic acid), GA (gallic acid), PA (protocatechuic acid), *p*-OH-BA (*p*-hydroxybenzoic acid), CA (caffeic acid), *p*-CoA (*p*-coumaric acid), SA (sinapic acid), EA (ellagic acid), D3G (delphinidin-3-glucoside), C3G (cyanidin-3-glucoside), C3R (cyanidin-3-rutinoside), P3G (pelargonidin-3-glucoside), Pe3G (peonidin-3-glucoside).

Table S13. Relations of several evaluation parameters of the nutraceutical mixtures with edible flowers evaluated under the storage conditions at 40 °C in a thermostatic device.

T	f-ABTS	f-DPPH	sb-ABTS	sb-DPPH	ib-ABTS	ib-DPPH	t-ABTS	t-DPPH	ACW	ACL	IAC
f-TPC	0.5376	0.6928	*	*	*	*	*	*	*	*	*
sb-TPC	*	*	0.4476	0.5771	*	*	*	*	*	*	*
ib-TPC	*	*	*	*	-0.2285	-0.3886	*	*	*	*	*
t-TPC	*	*	*	*	*	*	-0.2661	0.0826	-0.2208	-0.3175	-0.2711
f-E	-0.9817	-0.6208	*	*	*	*	*	*	*	*	*
sb-E	*	*	0.7811	-0.0165	*	*	*	*	*	*	*
ib-E	*	*	*	*	0.2506	0.2954	*	*	*	*	*
t-E	*	*	*	*	*	*	-0.3240	-0.4650	-0.4346	0.3282	-0.1930
f-C	0.2684	0.4955	*	*	*	*	*	*	*	*	*
sb-C	*	*	0.1802	0.9403	*	*	*	*	*	*	*
ib-C	*	*	*	*	-0.0963	-0.0661	*	*	*	*	*
t-C	*	*	*	*	*	*	-0.4026	-0.0668	-0.3393	-0.5280	-0.4323
f-R	0.4864	0.6396	*	*	*	*	*	*	*	*	*
sb-R	*	*	-0.0390	0.7644	*	*	*	*	*	*	*
ib-R	*	*	*	*	0.0987	0.2984	*	*	*	*	*
t-R	*	*	*	*	*	*	0.3444	0.6453	0.3978	0.0767	0.3143
f-Q	0.7269	0.6332	*	*	*	*	*	*	*	*	*
sb-Q	*	*	0.4410	0.6166	*	*	*	*	*	*	*
ib-Q	*	*	*	*	0.9070	0.8251	*	*	*	*	*
t-Q	*	*	*	*	*	*	0.6539	0.3691	0.5862	0.7626	0.6943
f-NA	-0.6248	-0.5611	*	*	*	*	*	*	*	*	*
sb-NA	*	*	0.0775	0.4708	*	*	*	*	*	*	*
ib-NA	*	*	*	*	0.6648	0.5369	*	*	*	*	*
t-NA	*	*	*	*	*	*	0.0667	-0.1274	0.1312	-0.3947	-0.0501
f-GA	0.6504	0.6303	*	*	*	*	*	*	*	*	*

sb-GA	*	*	-0.4595	-0.7483	*	*	*	*	*	*	*
ib-GA	*	*	*	*	0.6442	0.5151	*	*	*	*	*
t-GA	*	*	*	*	*	*	0.9746	0.8580	0.9763	0.6768	0.9435
f-PA	0.1550	0.6954	*	*	*	*	*	*	*	*	*
sb-PA	*	*	0.6543	-0.2903	*	*	*	*	*	*	*
ib-PA	*	*	*	*	0.1187	0.0688	*	*	*	*	*
t-PA	*	*	*	*	*	*	-0.1670	-0.2877	-0.2841	0.4915	-0.0260
f-*p*-OH-BA	0.4279	0.6253	*	*	*	*	*	*	*	*	*
sb-*p*-OH-BA	*	*	0.0642	0.6699	*	*	*	*	*	*	*
ib-*p*-OH-BA	*	*	*	*	0.7339	0.5767	*	*	*	*	*
t-*p*-OH-BA	*	*	*	*	*	*	0.5452	0.7622	0.6222	0.0598	0.4683
f-CA	0.5136	0.7575	*	*	*	*	*	*	*	*	*
sb-CA	*	*	0.2201	0.1009	*	*	*	*	*	*	*
ib-CA	*	*	*	*	-0.0209	0.0511	*	*	*	*	*
t-CA	*	*	*	*	*	*	0.5129	0.7460	0.5037	0.5323	0.5548
f-*p*-CoA	-0.7010	-0.6178	*	*	*	*	*	*	*	*	*
sb-*p*-CoA	*	*	0.1898	0.7955	*	*	*	*	*	*	*
ib-*p*-CoA	*	*	*	*	0.0303	0.1447	*	*	*	*	*
t-*p*-CoA	*	*	*	*	*	*	-0.6297	-0.8318	-0.6962	-0.1770	-0.5636
f-SA	0.9161	0.6608	*	*	*	*	*	*	*	*	*
sb-SA	*	*	-0.3851	0.8968	*	*	*	*	*	*	*
ib-SA	*	*	*	*	-0.2293	-0.0902	*	*	*	*	*
t-SA	*	*	*	*	*	*	0.6382	0.8522	0.6945	0.2465	0.5877
f-EA	-0.2423	0.0523	*	*	*	*	*	*	*	*	*
sb-EA	*	*	-0.8495	0.3942	*	*	*	*	*	*	*
ib-EA	*	*	*	*	0.3269	0.3191	*	*	*	*	*
t-EA	*	*	*	*	*	*	0.1470	-0.1172	0.0382	0.6229	0.2515

t-TAC	*	*	*	*	*	*	0.8150	0.7227	0.7409	0.9971	0.8912
D3G	*	*	*	*	*	*	0.9557	0.8805	0.9146	0.9227	0.9886
C3G	*	*	*	*	*	*	-0.7305	-0.9139	-0.7384	-0.5784	-0.7392
C3R	*	*	*	*	*	*	0.9775	0.9039	0.9457	0.8864	0.9978
P3G	*	*	*	*	*	*	0.4731	0.6075	0.4108	0.7644	0.5718
Pe3G	*	*	*	*	*	*	-0.4420	-0.1066	-0.3877	-0.5065	-0.4592
Delphinidin	*	*	*	*	*	*	0.8076	0.8658	0.7618	0.8915	0.8687
Cyanidin	*	*	*	*	*	*	0.2412	-0.1049	0.2084	0.2291	0.2302
Pelargonidin	*	*	*	*	*	*	0.4031	0.2339	0.2906	0.8749	0.5241
Peonidin	*	*	*	*	*	*	*	*	*	*	*
Malvidin	*	*	*	*	*	*	0.7731	0.5569	0.6996	0.9125	0.8302

T (storage experiment provided in the thermostatic device at 40 °C), f (free), sb (soluble bound), ib (insoluble bound), TPC (total phenolic content), TAC (total anthocyanin content), E (epicatechin), C (catechin), R (rutin), Q (quercetin), NA (neochlorogenic acid), GA (gallic acid), PA (protocatechuic acid), *p*-OH-BA (*p*-hydroxybenzoic acid), CA (caffeic acid), *p*-CoA (*p*-coumaric acid), SA (sinapic acid), EA (ellagic acid), D3G (delphinidin-3-glucoside), C3G (cyanidin-3-glucoside), C3R (cyanidin-3-rutinoside), P3G (pelargonidin-3-glucoside), Pe3G (peonidin-3-glucoside).
